# Effects of Structure and Meaning on Cortical Tracking of Linguistic Units in Naturalistic Speech

**DOI:** 10.1162/nol_a_00070

**Published:** 2022-06-21

**Authors:** Cas W. Coopmans, Helen de Hoop, Peter Hagoort, Andrea E. Martin

**Affiliations:** Max Planck Institute for Psycholinguistics, Nijmegen, The Netherlands; Centre for Language Studies, Radboud University, Nijmegen, The Netherlands; Donders Institute for Brain, Cognition and Behaviour, Radboud University, Nijmegen, The Netherlands

**Keywords:** EEG, mutual information, compositionality, idioms, jabberwocky

## Abstract

Recent research has established that cortical activity “tracks” the presentation rate of syntactic phrases in continuous speech, even though phrases are abstract units that do not have direct correlates in the acoustic signal. We investigated whether cortical tracking of phrase structures is modulated by the extent to which these structures compositionally determine meaning. To this end, we recorded electroencephalography (EEG) of 38 native speakers who listened to naturally spoken Dutch stimuli in different conditions, which parametrically modulated the degree to which syntactic structure and lexical semantics determine sentence meaning. Tracking was quantified through mutual information between the EEG data and either the speech envelopes or abstract annotations of syntax, all of which were filtered in the frequency band corresponding to the presentation rate of phrases (1.1–2.1 Hz). Overall, these mutual information analyses showed stronger tracking of phrases in regular sentences than in stimuli whose lexical-syntactic content is reduced, but no consistent differences in tracking between sentences and stimuli that contain a combination of syntactic structure and lexical content. While there were no effects of compositional meaning on the degree of phrase-structure tracking, analyses of event-related potentials elicited by sentence-final words did reveal meaning-induced differences between conditions. Our findings suggest that cortical tracking of structure in sentences indexes the internal generation of this structure, a process that is modulated by the properties of its input, but not by the compositional interpretation of its output.

## INTRODUCTION

How the brain parses a continuous speech stream into discrete, hierarchically organized units of linguistic representation remains an important question in the neurobiology of language (Giraud & Poeppel, 2012; Martin, 2016, 2020; Meyer et al., 2020). A possible mechanism that the brain might use to extract linguistic information relies on phase alignment between neural activity and quasi-regular properties of the speech signal. This process, called *cortical tracking*, results from the tendency of neural systems to adjust to the timing of (quasi-)regular aspects of external stimuli, and has been argued to facilitate segmentation and parsing of continuous speech (for reviews, see Ding & Simon, 2014; Giraud & Poeppel, 2012; Kösem & van Wassenhove, 2017; Obleser & Kayser, 2019; Peelle & Davis, 2012; Rimmele et al., 2018; Schroeder & Lakatos, 2009; Zoefel & VanRullen, 2015).

Cortical tracking is well established for low-level aspects of the linguistic signal, which have clear correlates in the physical instantiation of speech (e.g., the speech envelope). Strikingly, recent work has shown that words and phrases, which are not clearly discernable in the speech signal and have to be internally constructed, are also cortically tracked (Ding et al., 2016; Ding, Melloni, et al., 2017). Moreover, these high-level linguistic properties influence lower-level speech processing, as shown by the fact that cortical tracking of the speech envelope is modulated by the listener’s knowledge of the language (Broderick et al., 2019; Di Liberto et al., 2018; Kaufeld et al., 2020).

These studies indicate that the inferred content of a signal affects the extent to which the brain tracks that signal (see also Keitel et al., 2018; Martin, 2020; ten Oever & Martin, 2021). What it is still elusive, however, is which aspects of *content* determine cortical speech tracking. In a recent paper, Kaufeld et al. (2020) showed that the neural signal aligns more strongly with periodically occurring linguistic units, such as syntactic phrases, when these contain meaningful information and are therefore relevant for linguistic processing. Specifically, cortical tracking of phrase structure was stronger for regular sentences than for control stimuli that were matched in terms of either lexical semantics (word lists) or both prosody and syntactic structure (jabberwocky sentences), suggesting that this neural response is driven by the compositional meaning of sentence structures. However, the difference between sentences and these control conditions can be described not only in terms of the output of compositional processing (i.e., the fact that sentence structures have a meaningful compositional interpretation), but also in terms of the factors that go into structural composition. To investigate which of these aspects of *linguistic content* affect cortical tracking of linguistic structure, the current electroencephalogram (EEG) study investigates cortical tracking of linguistic units (phrases, words, syllables) when these are embedded in stimuli that are parametrically varied in terms of the amount of linguistic information. These stimuli ranged from regular compositional sentences to structure-meaning divergent forms (idioms, syntactic prose), structures with reduced lexical-syntactic content (jabberwocky) and unstructured word lists. We thus test how the relationship between structure and meaning in spoken language affects cortical tracking of linguistic information.

### Cortical Tracking of Linguistic Structure

Low-frequency cortical activity closely tracks the amplitude envelope of the speech signal (Ahissar et al., 2001; Doelling et al., 2014; Gross et al., 2013; Kayser et al., 2015; Keitel et al., 2017, 2018; Luo & Poeppel, 2007). Because the low-frequency periodicity of the speech envelope correlates with the syllable rate (i.e., in the theta band), it has been argued that cortical activity in this frequency range tracks syllable-sized linguistic units (Giraud & Poeppel, 2012; Luo & Poeppel, 2007; Peelle & Davis, 2012; Poeppel & Assaneo, 2020). However, speech contains temporal regularities at multiple timescales; high-level linguistic units, such as syntactic phrases, also exhibit quasi-regular temporal structure, yet only a small number of studies have investigated cortical tracking of phrase structure.

A main method to study tracking of abstract structure has relied on careful control of the presentation rate of linguistic information, whereby this information is *frequency tagged*. The idea behind this approach is that when information is presented repeatedly at a specific frequency, the neural response to that type of information synchronizes with its presentation rate. In a series of MEG/EEG (magnetoencephalography/electroencephalography) studies, Ding and colleagues have shown that neural activity becomes phase-locked to the presentation rate of phrases and sentences, even though these abstract units are not physically discernable in the auditory signal itself (Blanco-Elorrieta et al., 2020; Ding et al., 2016; Ding, Melloni, et al., 2017; Getz et al., 2018; Makov et al., 2017; Sheng et al., 2019). Such phase-locked responses are found only if the input can be grouped into phrases, showing that they are based on linguistic knowledge, not acoustic information (Ding et al., 2016; Martin & Doumas, 2017). And while it has been disputed that what is tracked is really abstract structure rather than lexical semantics (Frank & Yang, 2018), recent studies have shown that lexical accounts cannot fully explain the data (Burroughs et al., 2021; Jin et al., 2020).

These frequency-tagging studies are very artificial because they rely on synthesized speech that is isochronously presented, but similar effects are reported in studies with more naturalistic materials. In one such study by Keitel et al. (2018), participants listened to naturally spoken sentences that were embedded in noise, after which they had to perform a comprehension task. All sentences were annotated for the occurrence of phrases, words, and syllables, yielding linguistically relevant frequency bands that were specific for their stimulus materials. Within each frequency band, speech tracking was quantified through mutual information between the speech envelope and neural activity. At the timescale of words and phrases, tracking was stronger for correctly comprehended than for incorrectly comprehended sentences, showing that speech tracking in these frequency bands is related to successful language comprehension.

Using a similar approach, Kaufeld et al. (2020) presented participants with naturally spoken stimuli in three conditions: regular sentences, jabberwocky sentences (i.e., same prosody and structure, but different lexical content), and word lists (i.e., same lexical content, but different structure and prosody). Backward versions of all stimuli were used to control for acoustic differences. At the phrasal timescale, speech tracking was stronger for regular sentences than for both jabberwocky sentences and word lists, while these differences were absent in the acoustic control conditions. These findings thus show that the brain is more attuned to phrases when they contain meaningful information and are therefore relevant for language comprehension (Kaufeld et al., 2020). In particular, the fact that phrase-level speech tracking is stronger for sentences than for jabberwocky suggests that this response is modulated by the semantic content of phrases (see also Brennan & Martin, 2020; Martin, 2020; Martin & Doumas, 2017).

It is still an open question, however, whether *semantic content* should be interpreted as lexical-semantic content—the fact that sentences are structured sequences composed of real words—or rather, compositional-semantic content—the fact that these real words in sentences compose into meaningful constituents. The most prominent difference between regular and jabberwocky sentences is that the former contain real content words, which are replaced by pseudowords in jabberwocky sentences. Real words and pseudowords differ in both semantic and lexical-syntactic content, with the latter strongly affecting linguistic structure building (e.g., Hagoort, 2005, 2017; Matchin & Hickok, 2020). It is thus possible to interpret the difference in phrase-level speech tracking between sentences and jabberwocky in two ways: Either it reflects the fact that words in sentences can be composed into meaningful constituents (i.e., reflecting the *outcome* of structure building; Kaufeld et al., 2020), or it reflects the fact that the lexical-syntactic information carried by content words allows words in sentences to be easily composed in the first place (i.e., reflecting the *input* to structure building). In the latter case, these findings reflect the brain’s attempt to build a structural representation of the linguistic input, regardless of its interpretation. The present study aims to tease apart these two possibilities.

### Background of the Present Study

We contrast regular sentences, whose meaning is compositionally derived from their structure and lexical components, with stimuli in which the mapping between structure and meaning is less transparent. If it is indeed the case that phrase-level speech tracking is driven by the structure-meaning correspondence of sentences, the tracking response should be stronger for sentences than for controls that are divergent in their structure-meaning relationship. As examples of the latter, we used one naturally occurring stimulus (idioms) and one artificial stimulus (syntactic prose), both of which contain the same structural and lexical-semantic information as regular (compositional) sentences, but are putatively less compositional in the sense that their meaning does not derive fully from a combination of their structure and lexical components. Parametrically reducing the amount of linguistic information, we also included jabberwocky sentences and unstructured word lists.

We note that compositional processing is not an all-or-none phenomenon (Baggio, 2021; Titone & Connine, 1999), and idioms and syntactic prose are not processed entirely non-compositionally. However, a compositional analysis of the sentences in these conditions either does not yield a sensible interpretation (syntactic prose) or does not yield the intended interpretation (idioms). We therefore assume that compositional processes will be overall less engaged in the comprehension of idioms and syntactic prose than in the comprehension of regular sentences.

Idioms are conventionalized co-occurrence restrictions whose figurative meaning must be learned (Cacciari, 2014; Cacciari & Glucksberg, 1991; Jackendoff, 1995, 2017). They adhere to basic grammatical rules but are semantically idiosyncratic: The figurative meaning of idioms is not fully derived from a semantic composition of their component parts (Cacciari & Glucksberg, 1991; Jackendoff, 1995, 2017; Sprenger et al., 2006). As an example, consider the Dutch idiom *een vinger aan de pols houden* (literally, “to keep a finger on the wrist”), whose figurative meaning is “to check whether everything goes right.” Clearly, this figurative meaning is non-compositional and conventionalized, but in terms of structure the idiom is not an unanalyzed whole. The idiom is a verb phrase whose verb inflects in the past tense in the same way it does in regular sentences (i.e., as in English, *houden* “to keep” is irregular, inflecting to *hield* “kept” in the past tense), and it has the regular argument structure of the verb *houden* “to keep,” which is used ditransitively and can be modified by adverbs in the usual way.

The idea that idioms contain regular syntactic structure is supported by evidence from language processing, which shows that the structure of idioms is accessed in both comprehension and production (Cutting & Bock, 1997; Konopka & Bock, 2009; Peterson et al., 2001; Sprenger et al., 2006). This structure is linked to the idiom’s meaning in a highly idiosyncratic way, but language users who process the idiom in real time cannot know this beforehand and will therefore initially attempt to derive its interpretation compositionally. Behavioral experiments show that while effects of compositionality can be found in the early stages of idiom comprehension, literal processing can to some extent be terminated after the phrase or sentence is recognized as being an idiom, at which point its idiomatic meaning is retrieved from semantic memory (Cacciari, 2014; Cacciari & Corradini, 2015; Cacciari & Tabossi, 1988; Holsinger & Kaiser, 2013; Libben & Titone, 2008; Peterson et al., 2001; though see Smolka et al., 2007). Evidence from electrophysiological brain recordings also suggests that compositional processes can be interrupted in the comprehension of idioms (Canal et al., 2017; Rommers et al., 2013; Vespignani et al., 2010). We therefore consider idioms suited to serve as experimental sentences whose meaning is not fully derived from their component parts. These effects of compositionality might not be apparent immediately (i.e., before the idiom recognition point), but we suspect that compositional processes will be overall less engaged for idioms than for regular sentences.

In syntactic prose, real words are used to construct syntactically correct but nonsensical sentences (e.g., Bastiaansen & Hagoort, 2015; Kaan & Swaab, 2002; Marslen-Wilson & Tyler, 1980; Mazoyer et al., 1993). As an example, consider the Dutch sentence *een prestatie zal het concept naar de mouwen leiden*, which translates as “an achievement will lead the concept to the sleeves.” This sentence adheres to the rules of Dutch syntax, including constraints on word order and argument structure, but a compositional analysis of the sentence does not yield an interpretation that makes sense. Not many studies have investigated the brain processes involved in comprehending syntactic prose, but one relevant study found increased EEG gamma-band power for regular sentences compared to syntactic prose (Bastiaansen & Hagoort, 2015). Notably, two other EEG studies reported similar effects in the gamma band when comparing regular sentences and idioms (Canal et al., 2017; Rommers et al., 2013), tentatively suggesting that the contrast between sentences and both idioms and syntactic prose affects similar neurocognitive processes.

In addition to these two conditions, we also used jabberwocky sentences and word lists (see also Kaufeld et al., 2020). With these five conditions in total (see examples in Table 1), our design parametrically varies the amount of linguistic information present in the stimuli. All conditions except jabberwocky sentences contained real content words, and all conditions except word lists had the same syntactic structure. Moreover, for all syntactically structured conditions, a compositional interpretation can be derived. However, a compositional combination of the words in idioms does not yield their figurative meaning, a compositional combination of the words in syntactic prose does not yield a coherent semantic interpretation, and a compositional combination of the (pseudo)words in jabberwocky sentences is underspecified. In other words, regular sentences differ from the other syntactically structured conditions not in whether they allow compositional processing in principle, but in whether a compositional combination of the structure and the lexical components yields a straightforward meaningful interpretation.

**Table 1.** Dutch example stimuli of all five conditions

**Condition**	**Stimulus**	**Lexical semantics**	**Syntactic structure**	**Meaningful compositional interpretation**
**Sentence**	*De jongen gaat zijn zusje met haar huiswerk helpen.*	X	X	X
	the boy will his sister with her homework help			
	“The boy will help his sister with her homework.”			
**Idiom**	*De directie zal een vinger aan de pols houden.*	X	X	
	the directorate will a finger on the wrist keep			
	Literal: “The directorate will keep a finger on the wrist.”			
	Figurative: “The directorate will check whether everything goes right.”			
**Syntactic prose**	*Een prestatie zal het concept naar de mouwen leiden.*	X	X	
	an achievement will the concept to the sleeves lead			
	“An achievement will lead the concept to the sleeves.”			
**Jabberwocky**	*De jormen gaat zijn lumse met haar luisberk malpen.*		X	
	the jormen will his lumse with her luisberk malp			
	“The jormen will malp his lumse with her luisberk.”			
**Word list**	*De gaat jongen zusje huiswerk zijn haar helpen met*	X		
	the will boy sister homework his her help with			

*Note*. English translations are provided above. Only the underlined words in the idiom stimulus are part of the conventionalized idiom.

### The Present Study

Participants listened to spoken stimuli in these conditions while their EEG was recorded. We quantified cortical tracking between the speech envelopes and the EEG data by means of *mutual information* (MI), which is an information-theoretic measure that quantifies the statistical dependence between two random variables (Cogan & Poeppel, 2011; Gross et al., 2013; Ince et al., 2017; Kayser et al., 2015; Keitel et al., 2017). MI was computed in three frequency bands, corresponding to the occurrence of phrases (1.1–2.1 Hz), words (2.3–4.7 Hz), and syllables (3.4–4.9 Hz) in our stimuli (Kaufeld et al., 2020; Keitel et al., 2018). Following previous research, we controlled for spectral differences between sentences and word lists by including backward versions of both stimuli. These backward versions preserve many of the spectral properties of their forward version (especially rhythmic components) but are unintelligible (Gross et al., 2013; Kaufeld et al., 2020; Keitel et al., 2017; Park et al., 2015).

We were particularly interested in the coherence between speech and EEG in the phrase frequency band. For this measure of phrase-level speech tracking we consider two possibilities. If it is affected by the extent to which a compositional analysis of the input yields a meaningful structural representation, we expect higher MI for regular sentences than for all other conditions. Instead, if phrase-level speech tracking reflects the construction of a structural representation regardless of its compositional interpretation, we do not expect MI for regular sentences to differ from MI for idioms and syntactic prose. Yet, we do predict MI to be higher for regular sentences than for jabberwocky and word lists, because the latter two contain less information based on which a structural representation can be constructed (i.e., cues from argument structure, word order).

## MATERIALS AND METHODS

### Participants

We recruited 40 participants (30 female, average age = 24.6 years, age range = 19–31 years) from the participant pool of the Max Planck Institute for Psycholinguistics. All participants were right-handed native speakers of Dutch, who reported normal hearing and did not have a history of language impairment. After receiving information about the experimental procedures, participants gave written informed consent to take part in the experiment, which was approved by the Ethics Committee of the Faculty of Social Sciences at Radboud University Nijmegen. They were reimbursed for their participation. After preprocessing, we excluded two participants due to low numbers of artifact-free trials. The analyses reported are based on a sample of 38 participants.

### Materials

#### Experimental items

An example of one stimulus item for each condition is given in Table 1. The Sentence condition contained sentences with compositional meaning, which is derived from a combination of the word meanings and their structural combination. To give an example of a translated stimulus item, the meaning of *The boy will help his sister with her homework* is a function of the meaning of the individual words and the syntactic structure of the sentence. The structure was the same for all syntactically structured conditions (i.e., Sentence, Idiom, Syntactic prose, and Jabberwocky), which start with a noun phrase (NP) and an auxiliary verb (e.g., *The boy will* …), followed by a verb phrase consisting of an NP, a prepositional phrase (PP), and a non-finite lexical verb (V), which is phrase-final in Dutch (e.g., … *help his sister with her homework*).

For the Idiom condition we selected a set of commonly used and well-known Dutch idioms that had the same NP-PP-V structure. The majority of these idioms were selected from stimulus lists shared by Hubers et al. (2018) and Rommers et al. (2013). The idioms were embedded in carrier sentences by the addition of a sentence-initial NP and an auxiliary verb, which are not part of the conventionalized structure. We only analyzed those idioms that were known to the participants. Idiom knowledge was established for each participant by means of a post-experiment questionnaire (see the section Idiom Knowledge Test). Syntactic prose sentences are grammatically well-formed and contain real words, but these are difficult to compose into a coherent semantic representation. The stimulus sets in both the sentence condition and the syntactic prose condition were matched with the idioms on the total number of syllables and on the lexical frequency of the content words (frequencies extracted from the SUBTLEX-NL database of Dutch word frequencies; Keuleers et al., 2010). Jabberwocky sentences were generated with the Wuggy pseudoword generator (Keuleers & Brysbaert, 2010), which generates pseudowords that obey the phonotactic constraints of Dutch. We created jabberwocky versions of all items in the sentence condition by substituting each content word with a pseudoword that was matched in number of syllables, subsyllabic structure, and syllable transition frequency. The function words (auxiliaries, determiners, prepositions, pronouns) were kept the same, allowing for the construction of the same syntactic structure with a compositional interpretation. Items in the Word list condition contained the same words as those in the corresponding sentence item, but were scrambled in such a way that no syntactic combinations could be formed.

We created 85 stimuli for all conditions, of which the first five served as practice trials, which were not analyzed. Only the idiom condition had 90 items, which allowed us to preserve roughly the same number of trials as in the other conditions after excluding unknown idioms.

#### Audio recordings

The stimuli were recorded in a sound-attenuated booth by a female native speaker of Dutch (sampling rate = 44.1 kHz (mono), bit depth = 16). After recording, the intensity of all stimuli was scaled to 70 dB in Praat (Version 6.1.02; Boersma & Weenink, 2019). Backward stimuli for the Sentence and Word list conditions were created by reversing each stimulus recording in Praat.

**Figure 1.**
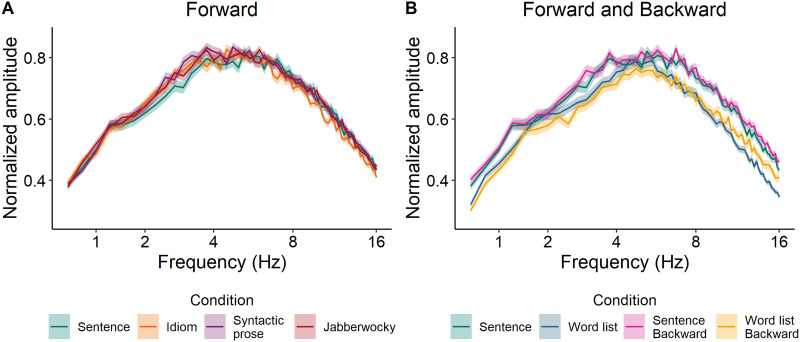
Modulation spectra of the forward versions of all conditions, computed following the procedure described in Ding, Patel, et al. (2017). Backward versions of sentences and word lists were included because of the differences between the forward versions of these two conditions.

Figure 1 shows the modulation spectra of all Forward conditions as well as the Backward version of sentences and word lists. These figures indicate that all Forward conditions are prosodically very similar (Figure 1A), except for the word list condition (Figure 1B), which deviates from the sentence condition at several frequencies. (See Supplementary Information S1. Supporting Information can be found at https://doi.org/10.1162/nol_a_00070.) While not ideal, we believe that this prosodic difference between stimuli with regular syntactic structure and those without structure is inherent in the contrast between these conditions. Because our main interest is the comparison between the syntactically structured conditions (Sentence vs. Idiom, Syntactic prose, Jabberwocky), it is important that these conditions do not systematically differ in acoustic properties.

### Annotations

**Figure 2.**
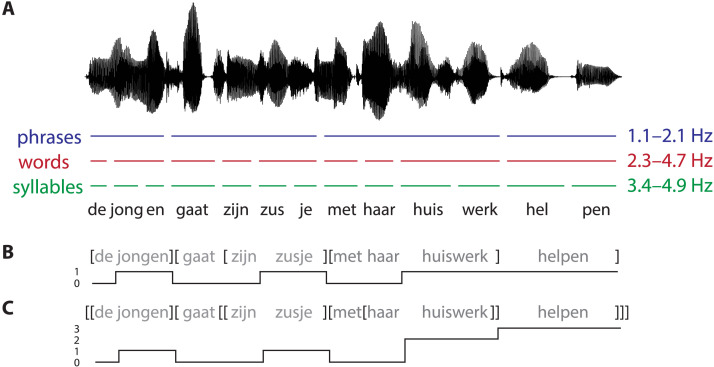
Three different annotations of linguistic structure for the Dutch translation of the sentence *the boy will help his sister with her homework*. (A) Schematic illustration of the three different timescales of the linguistic units of information (phrases, words, and syllables) contained in the sentence. From the annotation of these timescales, we derived frequency bands for each linguistic unit. (B) Phrase-level annotation, where words that integrate a phrase are coded as 1 for their entire duration, while all other words are coded as 0 (*bracket presence*). (C) Phrase-level annotation, where the value assigned to each word corresponds to the number of phrases that the word integrates (*bracket count*).

We manually annotated the forward recordings in Praat (Boersma & Weenink, 2019) with respect to the presence of phrases, words, and syllables. Specifically, for each stimulus we annotated the position in the recording where a linguistic unit ends (Figure 2A). For both words and syllables, this corresponds to the boundary between successive units. For phrases this corresponds to the position of closing phrase boundaries. For example, in [*de jongen*] [*gaat* [*zijn zusje*] [*met haar huiswerk*] *helpen*] the closing bracket denotes the offset of a phrase whose onset is denoted by the corresponding opening bracket. As word lists by definition do not contain phrases, we marked “phrases” in these stimuli by annotating the offsets of the words that are at positions of closing phrase boundaries in the corresponding sentence item. In the example sentence above, phrases are closed after the second, fifth, eighth, and ninth word, leading to the following phrase annotation for the corresponding word list: [*de gaat*] [*jongen* [*zusje huiswerk*] [*zijn haar helpen*] *met*]. Converting the onsets and offsets of these annotations to frequencies resulted in the following frequency bands: 1.1–2.1 Hz for phrases, 2.3–4.7 Hz for words, and 3.4–4.9 Hz for syllables.

To provide additional evidence that our results index cortical tracking of abstract (syntactic) information, rather than mere acoustic differences between the conditions, we performed an additional MI analysis in which the speech stimuli were replaced by abstract versions of these stimuli in which we only encoded phrase-structure information (Brodbeck, Presacco, & Simon, 2018; Kaufeld et al., 2020). For each forward stimulus, we marked all time points corresponding to phrase-final words with a 1 and marked all other time points with a 0 (*bracket presence*; Figure 2B). Phrase-final words are those words at which syntactic and/or semantic composition can take place. For example, the time points corresponding to the underlined words in the sentence [*de jongen*] [*gaat* [*zijn zusje*] [*met haar huiswerk*] *helpen*] (i.e., *boy*, *sister*, *homework*, *help*) were marked by a 1, because they close syntactic phrases, while all other time points were marked by a 0. Again, annotating phrase-final words in word lists is impossible, so we marked phrases in the same way as described above, marking time points corresponding to words with a 1 if these words are in a position that indexes a phrase-final word in the corresponding sentence.

These abstract annotations of bracket presence are actually insufficient to represent phrase structure, because sentences are hierarchically embedded structures rather than linearly concatenated phrases. To represent this property, we incorporated *bracket count* as yet another type of abstract annotation (Brennan & Martin, 2020; Brennan et al., 2012; Brennan et al., 2016; Nelson et al., 2017), which is correlated with bracket presence but contains more detailed syntactic information. This variable counts the number of phrases that are completed at a particular word (derived from bottom-up tree traversal), corresponding to the closing brackets in [[*de jongen*] [*gaat* [[*zijn zusje*] [*met* [*haar huiswerk*]] *helpen*]]]. The value assigned to each word for its entire duration corresponds to the number of phrases that the word integrates (Figure 2C).

### Experimental Design

Participants listened to all stimuli in all seven conditions, which were presented in a block design. The order in which the seven blocks were presented was pseudo-randomized, with the following constraints: The two backward conditions were never presented in adjacent blocks, and the block with word lists and the block with idioms always preceded the block with sentences. Regarding the word lists, this presentation order was used to reduce the possibility that participants would project (their memory of) the phrase structure of the sentences onto the word lists. Regarding the idioms, this order was used to reduce the possibility that participants would try to derive a compositional analysis of their meaning. Within each block, the order of the items was randomized.

### Procedure

Participants were individually tested in a soundproof booth. They were instructed to attentively listen to the audio, which was presented over loudspeakers, while looking at a fixation cross displayed at the center of the screen. After each trial, participants had to advance to the next trial by pressing a button. They were allowed to take short breaks between blocks. The EEG experiment lasted approximately 60–70 min and was followed by an idiom knowledge test.

### Idiom Knowledge Test

The EEG experiment was followed by a digital questionnaire in which participants were asked to indicate whether they knew the figurative meaning of the idioms that were presented in the experiment. For each idiom, they had to indicate this on a keyboard. If they answered “yes,” they had to type the meaning using the keyboard. If they answered “no,” they were asked to indicate what they thought the meaning could be. Idioms were rated as *known* when the participant answered “yes” and gave a correct description of the meaning of the idiom. For each participant, we included only idioms rated as known into subsequent analyses. On average, participants knew 78 of the 90 idioms (86.7%, range = 64–90).

### Speech Preprocessing

**Figure 3.**
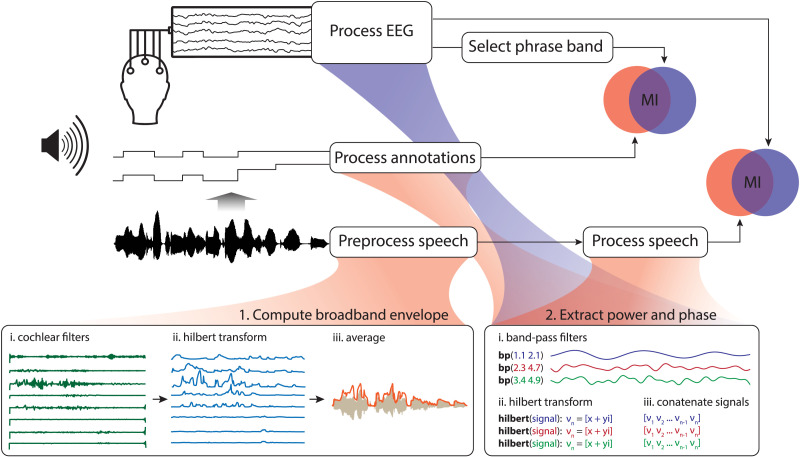
Visual representation of the analysis pipeline.

The speech envelope is the acoustic power of the speech signal at a given time in a given frequency range. Here, we estimated the broadband speech envelope by averaging across all ranges, following the procedure described in Chandrasekaran et al. (2009) and adopted by subsequent studies (Gross et al., 2013; Kaufeld et al., 2020; Kayser et al., 2015; Keitel et al., 2017). Using the Chimera toolbox (Smith et al., 2002), we band-pass filtered the auditory signal into 8 frequency bands between 100–8000 Hz (third-order Butterworth filter, forward and reverse), such that the bands spanned equal widths on the cochlear frequency map (1.i in Figure 3). The cutoff frequencies of the bands (in Hz) were: 100, 228, 429, 743, 1233, 2000, 3198, 5071, and 8000. We computed the Hilbert transform of the signal in each of these frequency bands and took the absolute value as an estimate of the narrowband envelope (1.ii in Figure 3). We downsampled each narrowband speech envelope to 150 Hz, and averaged across all 8 bands to derive the broadband speech envelope (1.iii in Figure 3).

### EEG Recording and Preprocessing

The EEG was recorded using an MPI custom actiCAP 64-electrode montage (Brain Products, Munich, Germany), of which 59 electrodes were mounted in the electrode cap (see Supplementary Information S2 for electrode layout). Eye blinks were registered by one electrode below the left eye, and eye movements were registered by two electrodes, placed on the outer canthi of both eyes. One electrode was placed on the right mastoid, the reference electrode was placed on the left mastoid and the ground was placed on the forehead. The EEG signal was amplified through BrainAmp DC amplifiers and referenced online to the left mastoid. The data were acquired at a sampling rate of 500 Hz, using a band-pass filter of 0.016–249 Hz.

Preprocessing was performed using the Fieldtrip toolbox (Oostenveld et al., 2011) in Matlab (Version 2016a). If channels were broken or showed heavy drifts, they were replaced by a weighted average of their neighbors. The data were then low-pass filtered at 50 Hz (36db/oct), re-referenced to the average of all electrodes and segmented into epochs ranging from the onset to the offset of the audio recording. We manually rejected trials that contained (movement) artifacts and trials in which an unknown idiom was presented (on a by-idiom, by-participant basis; based on the post-experiment questionnaire). We used independent component analysis (ICA; using ICA weights from a version of the data which was downsampled to 300 Hz and high-pass filtered at 1 Hz) to filter artifacts resulting from eye movements and steady muscle activity. Last, we automatically rejected epochs in which the difference between the maximum and minimum voltage exceeded 150 μV. In total, we excluded 9.2% of the data (range of averages across conditions = 6.4%–12.1%). Each EEG segment was downsampled to 150 Hz to match the sampling rate of the speech envelopes. The preprocessed data were then subjected to mutual information analysis.

### Mutual Information Analysis

To quantify cortical speech tracking in each frequency band, we computed MI between the band-limited Hilbert representations of the broadband speech envelope and the EEG signal (see Figure 3). In our experiment, MI measures the average reduction in uncertainty about the EEG signal given that the speech envelope (or annotation of syntax) is known, and can thus be used as a measure of the relatedness of the two signals (Ince et al., 2017). We followed the procedure described in Kaufeld et al. (2020), which involved the following steps for speech signals and EEG trials separately: First, each signal was band-pass filtered in the frequency bands of interest (Figure 3, 2.i), using third-order Butterworth filters (forward and reverse). We then extracted the complex components from each filtered signal using a Hilbert transform (Figure 3, 2.ii), whose real and imaginary parts were normalized separately using the copula normalization method developed by Ince et al. (2017). We derived instantaneous phase and power and concatenated the resulting signals from all trials (Figure 3, 2.iii). MI was computed for each electrode, participant, and condition separately, in the following way:

*MI*(EEG; Speech) = *H*(EEG) + *H*(Speech) − *H*(EEG, Speech)

Here, *H*(EEG) is the entropy of the (Hilbert representation of the) EEG signal, *H*(Speech) the entropy of the (Hilbert representation of the) broadband speech envelope, and *H*(EEG, Speech) their joint entropy. To accommodate speech-brain lag, we computed MI at five different lags, ranging from 60 to 140 ms, in steps of 20 ms. Statistical analysis was done on the average MI across all five lags.

The same steps were taken for the abstract stimuli, except that the band-pass filter was applied in the phrase frequency band only. MI was computed between the Hilbert representations of the abstract stimuli and the EEG signals corresponding to all forward conditions. For clarification, we use the term *speech tracking* to refer to MI computed between EEG and the speech envelopes, and *syntax tracking* to refer to MI computed between EEG and the abstract annotations of syntax.

### Statistical Analysis of MI Values

We fitted linear mixed-effects models (Baayen et al., 2008) to the log-transformed, trimmed (2.5% at both tails of the distribution of each condition) MI values in each frequency band and in a centroparietal cluster of electrodes (electrodes 1, 3, 4, 5, 8, 9, 10, 11, 28, 29, 30, 33, 35, 36, 37, 40, 41, 42, 43, based on Kaufeld et al., 2020; see Supplementary Information S2 for electrode layout) using lme4 (Bates et al., 2015) in R (R Core Team, 2021). In each frequency band we ran two separate models for the MI analysis between EEG and speech. The first model compared MI for Sentences to MI for Idioms, Syntactic prose, and Jabberwocky. This model contained the four-level factor Construction as fixed effect, which was treatment-coded with Sentence as the reference level. Participant was added as a random effect, which had a random intercept and Construction as random slope. Because we had backward versions of Sentences and Word lists, we compared Sentences to Word lists in a two-by-two analysis. This involved a second model with Structure (Sentence vs. Word list), Direction (Forward vs. Backward), and their interaction as fixed effects. Structure and Direction were deviation coded (−0.5, 0.5), and participant was added as random effect, with a random intercept and the interaction between Structure and Direction as random slope. This second model evaluates whether the MI difference between Sentences and Word lists in the forward version is different from the same difference in the backward version.

For the MI analyses in which the speech envelope was replaced by abstract annotations, we ran a model with the five-level factor Construction (Sentence, Idiom, Syntactic prose, Jabberwocky, Word list) as fixed effect. This model compared MI for Sentences to MI for Idioms, Syntactic prose, Jabberwocky, and Word lists. Construction was again treatment-coded with Sentence as the reference level. Participant was added as a random effect, which had a random intercept and Construction as random slope. In all analyses we evaluated whether adding a fixed effect increased predictive accuracy by comparing a model with that fixed effect to a model without that fixed effect using R’s anova() function.

### ERP Preprocessing and Analysis

To evaluate whether the different forward conditions were processed as intended, we compared the event-related potentials (ERPs) elicited by the sentence-final lexical verb in all syntactically structured conditions (i.e., Sentence, Idiom, Syntactic prose, and Jabberwocky). Word lists were not included because the lexical verbs in word lists were not sentence-final (see Table 1), due to which the ERP windows segmented around these verbs also contained activity evoked by the subsequent word. We were specifically interested in the N400, a negative-going ERP component that peaks between 300 and 500 ms after the onset of each content word and is sensitive to predictability and semantic congruency (Baggio & Hagoort, 2011; Kutas & Federmeier, 2011).

Because the segments corresponding to the N400 for these sentence-final verbs lasted beyond the offset of the audio recordings, they were not captured in the segments we used for MI analysis. We therefore used a separate preprocessing pipeline for the ERP analysis, in which the data were low-pass filtered at 40 Hz (36db/oct), re-referenced to the average of the left and right mastoid, and segmented into epochs ranging from −250 to 1,500 ms relative to the onset of the sentence-final verb in each audio recording. All other preprocessing steps were identical to those reported in the section EEG Recording and Preprocessing. In total, we excluded 4.8% of the data (range of averages across conditions = 4.1%–5.3%). Before statistical analysis, the EEG data were baseline-corrected using a 250 ms baseline window preceding the sentence-final verb.

For the N400 region of interest, we calculated the voltage in the centroposterior electrodes 3, 8, 9, 15, 27, 28, 35, 40, 41, 47 in a 300–500 ms time window after the onset of the sentence-final word, for each trial and each participant (based on Coopmans & Nieuwland, 2020, see Supplementary Information S2 for electrode layout). These voltage values were compared via a linear mixed-effects analysis (Baayen et al., 2008) in R (R Core Team, 2021). The mixed-effects model contained Construction as fixed effect, which was treatment-coded with Sentence as the reference level to which the conditions Idiom, Syntactic prose, and Jabberwocky were individually compared. We included participant as random effect, which had a random intercept and Construction as random slope. The models with and without Construction were compared with R’s anova() function.

## RESULTS

### Speech Tracking

**Figure 4.**
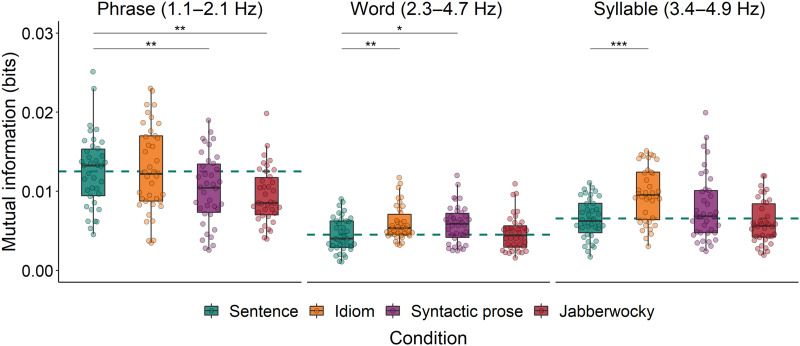
Mutual information between EEG and the speech envelopes of all syntactically structured conditions in the phrase, word, and syllable frequency bands. Drops reflect average per participant. The dashed horizontal line reflects the average of the Sentence condition. *p < 0.05, **p < 0.01, ***p < 0.001.

**Figure 5.**
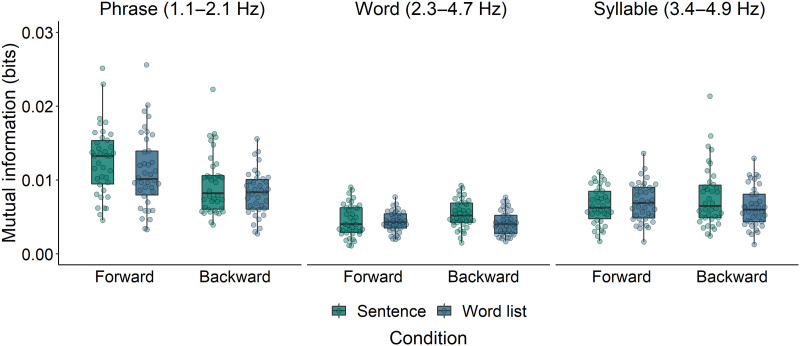
Mutual information between EEG and the speech envelopes of both forward and backward versions of Sentences and Word lists in the phrase, word, and syllable frequency bands. Drops reflect average per participant.

In the phrase frequency band, we ran two separate mixed-effects models. The first model evaluates whether MI is modulated by the type of Construction that was presented, comparing Sentences to the other syntactically structured conditions (Idioms, Syntactic prose, and Jabberwocky). Model comparison showed that Construction predicted MI (χ^2^ = 15.30, *p* = 0.002; see left panel of Figure 4). Specifically, MI was higher for Sentences than for both Jabberwocky and Syntactic prose, but not different from MI for Idioms (see Table 2 for the estimates of the fixed effects). The second model evaluated the interaction between Structure (Sentence vs. Word list) and Direction (Forward vs. Backward). Model comparison revealed that Sentences elicited higher MI than Word lists (χ^2^ = 4.19, *p* = 0.041; see left panel of Figure 5), and that Forward stimuli elicited higher MI than Backward stimuli (χ^2^ = 14.37, *p* < 0.001). The interaction was not significant (χ^2^ = 0.72, *p* = 0.40), which means that the difference between Sentences and Word lists was not solely driven by the linguistic differences between their forward versions and thus (at least partially) also reflects differences in acoustics. The estimates of the fixed effects of this second model are presented in Table 3.

**Table 2.** Fixed effects of the models that compare speech tracking (i.e., speech-brain MI) for Sentences to speech tracking for Idioms, Syntactic prose, and Jabberwocky

	**Estimate**	***SE***	***df***	***t* value**	***p* value**
**Phrase frequency band**					
Intercept	−4.77	0.07	37.5	−70.68	<0.001
Sentence-Idiom	−0.05	0.10	38.0	−0.52	0.61
Sentence-Syntactic prose	−0.30	0.10	38.0	−2.88	0.007
Sentence-Jabberwocky	−0.38	0.11	38.0	−3.36	0.002
**Word frequency band**					
Intercept	−5.82	0.09	37.6	−65.05	<0.001
Sentence-Idiom	0.31	0.10	37.9	3.08	0.004
Sentence-Syntactic prose	0.26	0.10	38.0	2.61	0.013
Sentence-Jabberwocky	0.02	0.13	38.0	0.15	0.88
**Syllable frequency band**					
Intercept	−5.44	0.08	37.5	−69.83	<0.001
Sentence-Idiom	0.40	0.09	38.3	4.58	<0.001
Sentence-Syntactic prose	0.12	0.10	38.0	1.15	0.26
Sentence-Jabberwocky	−0.11	0.08	38.0	−1.39	0.17

*Note*. The estimates are from three different models, corresponding to the phrase, word, and syllable frequency bands. *SE* = standard error; *df* = degrees of freedom.

**Table 3.** Fixed effects of the interaction models, which evaluate the effects of Structure (Sentence vs. Word list) and Direction (Forward vs. Backward) on speech tracking (i.e., speech-brain MI)

	**Estimate**	***SE***	***df***	***t* value**	***p* value**
**Phrase frequency band**					
Intercept	−5.00	0.04	37.8	−123.55	<0.001
Structure	−0.15	0.07	38.1	−2.07	0.046
Direction	−0.28	0.07	38.0	−4.10	<0.001
Structure*Direction	−0.12	0.14	38.2	−0.85	0.40
**Word frequency band**					
Intercept	−5.77	0.04	37.6	−161.66	<0.001
Structure	−0.11	0.06	38.2	−1.82	0.077
Direction	0.09	0.08	37.9	1.08	0.29
Structure*Direction	0.25	0.14	38.2	1.75	0.088
**Syllable frequency band**					
Intercept	−5.43	0.04	38.0	−135.37	<0.001
Structure	−0.13	0.09	37.8	−1.50	0.14
Direction	0.03	0.08	37.6	0.45	0.66
Structure*Direction	0.23	0.15	38.1	1.55	0.13

*Note*. The estimates are from three different models, corresponding to the phrase, word, and syllable frequency bands. *SE* = standard error; *df* = degrees of freedom.

In the word frequency band, the first model showed that Construction predicted MI (χ^2^ = 11.71, *p* = 0.008; see middle panel of Figure 4), but this effect was not driven by the same contrasts as the effect in the phrase frequency band. That is, MI was lower for Sentences than for both Idioms and Syntactic prose, but not different from MI for Jabberwocky (Table 2). The second model showed a marginal difference between Sentences and Word lists (χ^2^ = 3.70, *p* = 0.054; see middle panel of Figure 5), and no difference between Forward and Backward stimuli (χ^2^ = 0.33, *p* = 0.56). The interaction between Structure and Direction was not significant (χ^2^ = 2.95, *p* = 0.086; Table 3).

In the syllable frequency band, the first model again showed that Construction predicted MI (χ^2^ = 22.15, *p* < 0.001; see right panel of Figure 4). MI was lower for Sentences than for Idioms, but not different from MI for Syntactic Prose and Jabberwocky (Table 2). The second model showed no difference between Sentences and Word lists (χ^2^ = 0.98, *p* = 0.32; see right panel of Figure 5), nor between Forward and Backward stimuli (χ^2^ = 0.10, *p* = 0.76). The interaction between Structure and Direction was also not significant (χ^2^ = 2.33, *p* = 0.13; Table 3).

### Syntax Tracking

**Figure 6.**
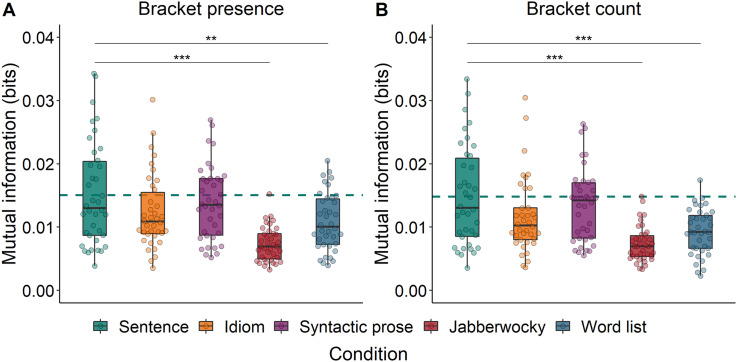
Mutual information between EEG and abstract annotations (bracket presence (A) and bracket count (B)) in the phrase frequency band. Drops reflect average per participant. The dashed horizontal line reflects the average of the Sentence condition. **p < 0.01, ***p < 0.001.

We then evaluated whether Construction (i.e., Sentence, Idiom, Syntactic prose, Jabberwocky, and Word list) predicted MI between the EEG signal and the abstract annotations of syntactic structure. When these annotations reflected bracket presence, Construction indeed predicted MI (χ^2^ = 35.98, *p* < 0.001; Figure 6A). MI was higher for Sentences than for both Jabberwocky and Word lists, but not different from MI for Idioms or Syntactic prose (see Table 4).

**Table 4.** Fixed effects of the models that compare syntax tracking (i.e., annotation-brain MI) for Sentences to syntax tracking for Idioms, Syntactic prose, Jabberwocky, and Word lists

	**Estimate**	***SE***	***df***	***t* value**	***p* value**
**Bracket presence**					
Intercept	−4.62	0.10	37.6	−47.17	<0.001
Sentence-Idiom	−0.19	0.13	37.5	−1.51	0.14
Sentence-Syntactic prose	−0.12	0.10	38.3	−1.23	0.23
Sentence-Jabberwocky	−0.74	0.13	37.8	−5.68	<0.001
Sentence-Word list	−0.35	0.12	37.9	−3.02	0.005
**Bracket count**					
Intercept	−4.64	0.10	37.6	−46.85	<0.001
Sentence-Idiom	−0.24	0.13	37.6	−1.83	0.076
Sentence-Syntactic prose	−0.13	0.11	38.1	−1.23	0.23
Sentence-Jabberwocky	−0.71	0.13	37.8	−5.30	<0.001
Sentence-Word list	−0.48	0.11	38.1	−4.25	<0.001

*Note*. The estimates are from two different models, corresponding to annotations reflecting respectively bracket presence and bracket count. *SE* = standard error; *df* = degrees of freedom.

The same pattern of results was found for the analysis of MI between the EEG signal and the annotations of bracket count, which differed across Constructions (χ^2^ = 34.12, *p* < 0.001; Figure 6B). MI was higher for Sentences than for Jabberwocky as well as for Word lists, but not different from MI for Idioms or Syntactic prose (see Table 4).

Overall, both analyses show that, at the frequency band corresponding to abstract phrase structure, the brain tracks the structure of sentences more strongly than the structure of both jabberwocky and word lists. It is interesting to note that the pattern of results is very similar for bracket count and bracket presence, suggesting that the more detailed syntactic information contained in the bracket count annotations does not add predictive accuracy with respect to phrase tracking (contrary to previous work, e.g., Brennan et al., 2016).

An anonymous reviewer rightly noted that the sentences in our conditions differ in co-occurrence frequency, with syntactic prose and jabberwocky sentences having lower transitional probabilities than regular sentences and idioms. We do not think this difference can account for our phrase-level effects, because it would predict a pattern of results that is different from what we found. First, it would predict no differences between syntactic prose and jabberwocky sentences, because they have similarly low transitional probabilities. Yet, these two conditions do elicit differences in cortical tracking of phrase structure. To test this, we repeated our linear mixed-effects analysis in the phrase frequency band, but with Syntactic prose as the reference level for the four-level factor Construction. When MI is computed between EEG and the abstract syntactic annotations, it is higher for Syntactic prose than for Jabberwocky, both when the annotations reflect bracket presence (β = −0.57, *SE* = 0.09, *t* = −6.32, *p* < 0.001; Figure 6A) and when they reflect bracket count (β = −0.62, *SE* = 0.09, *t* = −6.55, *p* < 0.001; Figure 6B). There were no differences between Syntactic prose and Jabberwocky in terms of speech tracking (β = −0.08, *SE* = 0.13, *t* = −0.59, *p* < 0.001). Second, it would predict differences between idioms and regular sentences, because the words in idioms are part of a fixed expression and therefore have high transitional probabilities. However, no such differences between sentences and idioms were found at the phrase level in either speech tracking (see the section Speech Tracking) or syntax tracking.

### ERPs to Sentence-Final Verb

The MI analyses showed no consistent differences in phrase tracking between Sentences and Idioms, whereas the difference between Sentences and Syntactic Prose was inconclusive (i.e., difference in speech tracking but no difference in syntax tracking). This absence of expected differences might indicate either that the brain does not track the syntactic structure of these stimuli differently (i.e., the conditions are perceived as being different, but this does not affect phrase tracking), or that the conditions were not processed as being very different). To evaluate the latter possibility, we compared the ERPs elicited by the sentence-final lexical verb in all syntactically structured conditions. The results show that the stimuli in the different conditions were processed as expected.

**Figure 7.**
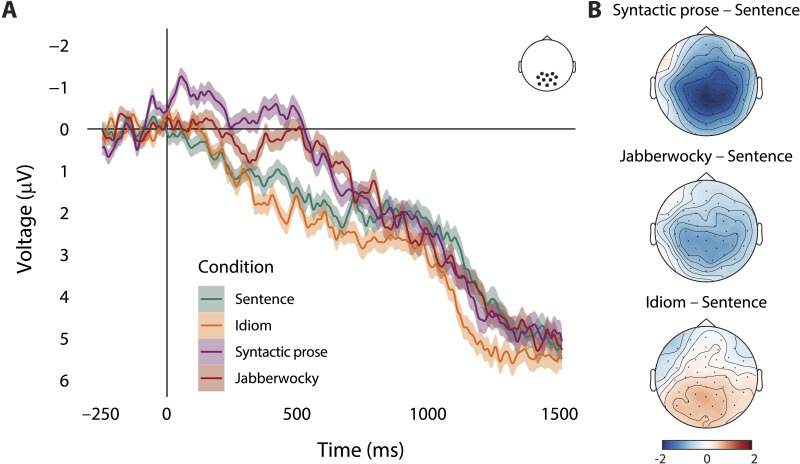
(A) Grand-average ERPs at the centroposterior cluster of electrodes, time-locked to the onset of the sentence-final verb in the four syntactically structured conditions. Negative voltage is plotted upwards, and color-shaded areas show the within-subjects standard error of the mean per time sample. (B) Topographical plots of the voltage differences between conditions in the 300–500 ms time window of interest.

As indicated by the sentence-final ERPs in Figure 7A, the variable Construction was associated with modulations of activity in the N400 region of interest (χ^2^ = 45.9, *p* < 0.001). The ERP elicited by sentence-final verbs in Sentences was less negative than the ERP elicited by sentence-final verbs in both Syntactic prose (β = −1.56, *SE* = 0.25, *t* = −6.37, *p* < 0.001) and Jabberwocky (β = −0.89, *SE* = 0.25, *t* = −3.51, *p* < 0.001), but more negative than the ERP elicited by sentence-final verbs in Idioms (β = 0.60, *SE* = 0.27, *t* = 2.26, *p* = 0.029). Note that the effects seem to start quite early, in particular for Syntactic prose (Figure 7A). This might have to do with the fact that the words preceding the verb in those stimuli are semantically odd (and thus elicit a strong N400), and with differences between conditions in the pre-verb parts in general. Figure 7B contains the topographical plots of the voltage differences in the 300–500 ms time window of interest.

## DISCUSSION

In this EEG study with naturally spoken stimuli, we investigated whether cortical tracking of phrase structure is modulated by the degree to which this structure is meaningful. Participants were presented with stimuli that contained different degrees of structural meaning. We measured tracking by computing mutual information between the EEG data and either the speech envelopes (speech tracking) or abstract annotations of syntax (syntax tracking). Both signals were filtered in the frequency band corresponding to the occurrence of phrases. These analyses showed overall stronger tracking of phrases in regular sentences than in stimuli with reduced lexical-syntactic content (jabberwocky) or without syntactic structure (word lists), but no consistent differences in phrase-level tracking between sentences and divergent stimuli that contained a combination of both structure and lexical meaning (idioms, syntactic prose). As analyses of sentence-final ERPs showed clear differences between the conditions in terms of their sentence-level meaning, we take these findings to suggest that cortical tracking of linguistic structure reflects the internal generation of that structure, whether it transparently maps onto semantic meaning or not.

### Effects of Composition in Processing Idioms and Syntactic Prose

We contrasted regular sentences to two semi-compositional conditions: Idioms and syntactic prose. We reasoned that compositional processes would be less engaged during the comprehension of idioms and syntactic prose (Canal et al., 2017; Peterson et al., 2001; Rommers et al., 2013; Vespignani et al., 2010), though there are several factors that likely influence the extent to which participants will try to derive a compositional interpretation from these sentences. Theories of idiom comprehension assume that before the idiom is recognized as being an idiomatic construction, standard literal processing is engaged (see Cacciari & Tabossi, 1988; Libben & Titone, 2008; Sprenger et al., 2006; Titone & Connine, 1999). As the idioms in our experiment were embedded in neutral carrier sentences, they could not be predicted from context, so the idiom recognition point might occur late. Sentence-final ERPs indicated that this was not too late to affect online comprehension: The N400 for sentence-final verbs was less negative in idioms than in regular sentences, suggesting that the idiom was recognized and retrieved before sentence offset. Nevertheless, any effects of compositionality are likely restricted to late time points, reducing the overall effect of compositionality on cortical tracking on phrase structure.

In addition, there are several differences within the group of idioms that might affect compositional processing. Many of the idiomatic constructions have variable slots that can be filled by compositional information (Jackendoff, 2017). The initial noun phrase in the idiom NP *door de vingers zien* (“to condone” NP), for instance, must be interpreted literally and will likely receive a compositional analysis. Relatedly, even in idioms that do not take variables, the literal meanings of the words are sometimes part of the idiom’s figurative meaning. For example, the idiom *de regels aan je laars lappen*, which literally means “to patch the rules to your boot” (figuratively: “to ignore the rules”) is actually about rules (though not about boots), so the NP *de regels* “the rules” will be processed literally. A question for future research is whether this variation within the class of idioms affects cortical tracking. We could not investigate this possibility, as it requires individual-item analyses that were impossible given the way MI was computed. Yet it would be interesting to see whether cortical tracking of linguistic units is affected by idiomatic variation, such as the transparency, decomposability, and syntactic flexibility of the idiom (see Cacciari, 2014; Cacciari & Glucksberg, 1991; Gibbs et al., 1989; Libben & Titone, 2008).

At the phrase timescale, we did not find a consistent difference between regular sentences and syntactic prose. While speech tracking was stronger for regular sentences than for syntactic prose (left panel of Figure 4), this difference was absent in measures of syntax tracking (Figure 6). Before we give our interpretation of these results, it is important to emphasize that the computation of speech tracking involves the speech envelope. While we filtered the signals in narrow frequency bands that were based on manual annotations of linguistic information in our stimuli (see the section Annotations), and while we checked for acoustic differences via analysis of the modulation spectra (Figure 1 and S1), we cannot rule out the possibility that any difference between the conditions in terms of speech tracking is driven by acoustic differences between the speech recordings. Such an effect would be in line with the fact that sentences and syntactic prose did not show differences in terms of syntax tracking, which is based on abstract annotations of syntax without any acoustic differences.

A possible reason for the lack of a consistent effect is that the difference between regular sentences and syntactic prose in terms of compositional processing is not the same at the phrase and sentence levels. At the sentence level, these conditions were clearly differentiable, as indicated by the ERP results. The N400 for sentence-final verbs was larger in syntactic prose than in regular sentences, showing that participants noticed the sentence-level semantic incongruency of syntactic prose. At the phrase-level, however, these conditions might impose similar demands on compositional processing, in particular if the type of composition is mostly syntactic. In contrast to previous research (Brodbeck et al., 2018; Kaufeld et al., 2020), much of the phrase-level compositional processing in our stimuli can be conceived of as mostly syntactic (i.e., containing a combination of a determiner and a noun) rather than semantic or conceptual. This applies even more strongly to our measure of syntax tracking, which is based on abstract annotations of phrase structure. These annotations do not distinguish between combinations like *the boat* and *red boat*, even though the latter phrase involves more semantic and conceptual composition. It is not unlikely that the presence of semantically impoverished combinations affects the overall degree of phrase-level tracking (i.e., making syntax tracking a less sensitive measure), especially in light of the finding that manipulations of lexical-semantic and conceptual composition affect measures of brain activity on top of the neural response to syntactic aspects of composition (e.g., Fedorenko et al., 2016, 2020; Kaufeld et al., 2020; Schell et al., 2017; Westerlund & Pylkkänen, 2014; Zhang & Pylkkänen, 2015).

### Sentences vs. Word Lists: Structure and Acoustics

We observed stronger phrase-level speech tracking for sentences than for word lists, and stronger tracking for forward stimuli than for backward stimuli (left panel of Figure 5). The difference between forward and backward speech has been reported before and is often related to differences in their intelligibility (Gross et al., 2013; Kaufeld et al., 2020; Park et al., 2015). However, other studies have failed to find such a relationship (Howard & Poeppel, 2010; Peña & Melloni, 2012; Zoefel & VanRullen, 2016), and it has been suggested that positive correlations between speech tracking and intelligibility are actually driven by the spectro-temporal properties of the unintelligible control condition (for discussion, see Kösem & van Wassenhove, 2017; Zou et al., 2019).

Contrasting with the results reported by Kaufeld et al. (2020), the MI difference between sentences and word lists did not differ across forward and backward versions of these conditions. We therefore cannot exclude the possibility that the difference between (forward) sentences and (forward) word lists reflects acoustic differences. Indeed, analysis of the modulation spectra shows that sentences were reliably different from word lists in terms of spectral properties (Figure 1B and Supplementary Information S1). However, the presence of acoustic differences does not necessarily negate the effect of syntactic differences. When the speech envelopes were replaced by abstract annotations of syntactic structure (bracket presence or bracket count), we found stronger MI for sentences than for word lists, presumably because these annotations do not reflect any syntactic information in word lists. This leaves open the possibility that syntactic structure did have an effect, but that it could not be detected in measures of speech tracking due to the masking effect of acoustic variance.

In order to find evidence for cortical tracking of phrase structure, we should find not only a difference in the forward condition (as a function of syntax), but also no difference in the backward condition. The latter might be quite difficult to obtain with our measures, because naturally produced sentences and word lists are acoustically quite different, and acoustic properties of the input can account for much of the variance in the neural response to speech (e.g., Brodbeck et al., 2018; Doelling et al., 2014). Moreover, the fact that syntactic information and suprasegmental modulations (e.g., prosodic phrases, intonation phrases) fluctuate at similar frequencies and both affect delta-band activity (Bourguignon et al., 2013; Ghitza, 2017; Meyer et al., 2017; Rimmele et al., 2021) makes it plausible that any structure-driven differences between sentences and word lists were partially masked by their acoustic differences. Supporting this possibility, analysis of spectral power in the phrase frequency band showed a bilateral distribution for all conditions (see Supplementary Information S3; see also e.g., Keitel et al., 2017; Molinaro & Lizarazu, 2018). This suggests that the neural signal in this frequency band is also affected by low-frequency modulations in suprasegmental information, which are present in both forward and backward recordings.

### Cortical Tracking of Lexicalized Structure

At the timescale of phrases, speech tracking was stronger for sentences than for jabberwocky and syntactic prose, but not different from idioms (left panel of Figure 4). In partial agreement with these results, syntax tracking was stronger for sentences than for jabberwocky and word lists, but not different from tracking for idioms and syntactic prose (Figure 6). Overall, cortical tracking of phrase structure seems to be enhanced for regular sentences compared to stimuli whose lexical-syntactic content is reduced (jabberwocky, word lists), but it is not consistently different from stimuli that contain both lexical content and syntactic structure (idioms, syntactic prose). This pattern of results is in line with the view that cortical tracking of syntactic structure reflects the generation of structure (Martin, 2020; Martin & Doumas, 2017, 2019; Meyer et al., 2020), whether this structure transparently maps onto a semantic interpretation or not.

Most current neurobiological models of language processing assume structure building to be a lexicalized process (Baggio, 2021; Hagoort, 2005, 2017; Martin, 2020; Matchin & Hickok, 2020). Words are associated with structures that are stored in the mental lexicon in the form of treelets. During language comprehension, these structures are combined to create the hierarchical structures of phrases and sentences. In this lexical-syntactic conception of structure building, the syntax determines which words can be combined, but the words themselves are the units of combination. As such, this process is affected by its input, in terms of both structure and lexical content. Input-wise, both jabberwocky and word lists are markedly different from sentences. Word lists contain content and function words but lack (cues to) syntactic structure, which means that adjacent words cannot be combined into phrasal units. Jabberwocky sentences are structured sequences that contain both function words and inflectional morphology, but they lack content words and therefore miss the information carried by their argument structure (e.g., the different relations in *saw* the book *on the table* and *put* the book *on the table*). The lexical-syntactic difference between regular and jabberwocky sentences thus explains why they elicit different degrees of phrase-level speech tracking: Structure-building processes are more weakly activated by lexically impoverished input. This idea is supported by evidence showing that lexical information affects neurocognitive measures of structure building (Burroughs et al., 2021; Fedorenko et al., 2016, 2020; Kaufeld et al., 2020; Matchin et al., 2019; Mollica et al., 2020).

Interestingly, in a recent study Mai and colleagues (2016) reported a different result, namely stronger delta-band speech tracking for stimuli containing pseudowords than stimuli containing real words. However, because their design was markedly different from ours, these findings do not necessarily contradict our interpretation. First, their pseudoword condition contained lists rather than sentences, so their two conditions differ in both structure (sentences vs. lists) and lexical-syntactic content (real words vs. pseudowords). Second, Mai et al. (2016) attribute their delta-tracking effect to the sound matching task they used, arguing that the semantic and syntactic content of real-word sentences facilitates the recognition of their phonological content. This reduces demands on phonological processing, as indexed by reduced speech tracking in the delta band. Because we instructed participants to listen for comprehension rather than to detect a specific sound, we think that our results are better accounted for in terms of lexical-syntactic structure building than in terms of phonological processing.

Idioms and syntactic prose are similar to sentences in lexical-syntactic structure, but they differ either in the extent to which their interpretation is compositionally derived from this structure (idioms) or in the extent to which a compositional interpretation of this structure makes sense (syntactic prose). Given the absence of consistent differences between these conditions, the interpretation most strongly supported by our data is that phrase-level tracking reflects the lexically-driven computation of syntactic structures in the service of semantic composition (Kaufeld et al., 2020; Martin, 2020; Martin & Doumas, 2017, 2019; Meyer et al., 2020). The computations involved in building hierarchical structure are most strongly activated by syntactically structured sequences of real words. Given the right input, these computations generate a compositional structure, whether the input can easily compose semantically or not. A similar idea has been proposed for the segmentation of complex word forms into stems and grammatical affixes (Marslen-Wilson, 2007). Behavioral and neuroimaging evidence shows that this morphophonological process is triggered by both real words and pseudowords, as long as they contain the diagnostic properties of inflectional affixes in English (Post et al., 2008; Tyler et al., 2005). To explain the lexical insensitivity of this process, Marslen-Wilson (2007, p. 180) argued that “without a decompositional analysis, the system cannot rule out the possibility that the pseudo-regular *trade* is actually the morpheme *tray* in the past tense, or that *snade* is the past tense of the potential real stem *snay*.” Analogously, without a decompositional analysis at the level of phrase structure, the system cannot rule out the possibility that “colorless green ideas sleep furiously” (Chomsky, 1957, p. 15) actually involves sleeping ideas or that *to kick the bucket* actually involves buckets being kicked. The conclusion that the compositional meaning of these forms is either semantically incoherent (syntactic prose) or not identical to their intended figurative meaning (idioms) can only be drawn after a compositional analysis has taken place. In a sense, then, the structure-building processes know *how* to build structure (i.e., adhering to syntactic rules, subcategorization restrictions), but not *what* is being built (i.e., whether a compositional analysis yields an interpretation that makes sense). Supporting this functional distinction between generation and interpretation, both behavioral and neurobiological evidence show a difference between sentences and both idioms and syntactic prose in terms of compositional meaning, but not in terms of syntactic structure building (Bastiaansen & Hagoort, 2015; Canal et al., 2017; Konopka & Bock, 2009; Peterson et al., 2001; Rommers et al., 2013; Vespignani et al., 2010).

### Effects of Composition on Word-Level Speech Tracking

At the timescale of words, speech tracking was stronger for both idioms and syntactic prose than for sentences (middle panel of Figure 4). These word-level effects might be related to the differential predictability of words in these conditions, as it has been shown that speech tracking is enhanced for unpredictable target words that are presented in low-constraining sentence contexts (Donhauser & Baillet, 2020; Molinaro et al., 2021). In these contexts, target words cannot be predicted by top-down mechanisms, so the brain might rely more strongly on the bottom-up input to ensure successful comprehension (Donhauser & Baillet, 2020; Molinaro et al., 2021). Words in syntactic prose are semantically odd and thus very unpredictable. On this account, the brain attunes more strongly to unexpected words (in syntactic prose), whose content can only be derived from a bottom-up analysis, than to expected words (in sentences), whose content can be predicted by top-down mechanisms, explaining the difference between syntactic prose and regular sentences in terms of word-level speech tracking.

ERP analysis showed that sentence-final verbs elicited a less negative N400 in idioms than in regular sentences, indexing facilitated activation or integration of the verb in idioms (e.g., Moreno et al., 2002; Rommers et al., 2013). This indicates that sentence-final verbs were more predictable in idioms than in sentences, in line with the behavioral literature (Cacciari et al., 2007; Cacciari & Tabossi, 1988). This predictability-related ERP difference is opposite to the difference between sentences and syntactic prose, which suggests that the difference between idioms and sentences in terms of word-level speech tracking (middle panel of Figure 4) does not have the same origin as the difference between syntactic prose and sentences. It is unclear why words would be tracked more closely in idioms, but one possibility is that participants were relatively more attuned to words in idioms because words are the linguistic unit on which participants have to rely to activate and retrieve the full idiom from memory (for discussion of the activation of properties of the individual words, see Cacciari, 2014; Cacciari & Tabossi, 1988; Hubers et al., 2021; Sprenger et al., 2006).

## CONCLUSION

Despite a constantly growing literature on cortical speech tracking, it is still unclear which aspects of high-level linguistic content drive neural activity into alignment with the speech signal. In this EEG study with naturally spoken stimuli, we used an experimental design with a parametric modulation of linguistic information, comparing compositional sentences to stimuli that diverged in terms of their relationship between structure and meaning (idioms, syntactic prose, jabberwocky, word lists). We found that the brain tracks syntactic phrases more closely in regular sentences than in stimuli whose lexical-syntactic content is reduced, but we found no consistent differences in phrase tracking between sentences and stimuli that contained a combination of both syntactic structure and lexical content. These findings refine a recent account of cortical speech tracking, which holds that it indexes the generation of linguistic structure (Martin & Doumas, 2017, 2019; Meyer et al., 2020). Specifically, they suggest that phrase-level speech tracking is modulated by the lexical-syntactic properties of the input to structure building, not by the compositional interpretation of its output. This is in line with neurobiological models of language processing in which structure building is a lexicalized process.

## ACKNOWLEDGMENTS

We thank Greta Kaufeld for helpful suggestions on the Mutual Information analysis and Eva Poort for the recordings of her voice.

## FUNDING INFORMATION

Andrea E. Martin, Nederlandse Organisatie voor Wetenschappelijk Onderzoek (https://dx.doi.org/10.13039/501100003246), Award ID: 016.Vidi.188.029. Peter Hagoort, Nederlandse Organisatie voor Wetenschappelijk Onderzoek (https://dx.doi.org/10.13039/501100003246), Award ID: 024.001.006. Andrea E. Martin, Max-Planck-Gesellschaft (https://dx.doi.org/10.13039/501100004189), Award ID: Lise Meitner Research Group.

## AUTHOR CONTRIBUTIONS

**Cas W. Coopmans**: Conceptualization; Data curation; Formal analysis; Investigation; Methodology; Project administration; Validation; Visualization; Writing – original draft; Writing – review & editing. **Helen de Hoop**: Conceptualization; Methodology; Writing – review & editing. **Peter Hagoort**: Conceptualization; Methodology; Writing – review & editing. **Andrea E. Martin**: Conceptualization; Methodology; Supervision; Writing – review & editing.

## TECHNICAL TERMS

Cortical speech tracking: The brain response related to slow fluctuations in the speech stimulus.
Speech envelope: Changes in the amplitude of a speech stimulus over time.
Jabberwocky sentence: A structurally coherent and prosodically natural sentence in which all content words are replaced by pseudowords.
Compositional meaning: The meaning of an expression that is a function of the meanings of its parts and their mode of combination.
Idiom: A more or less fixed phrase or expression with a figurative, non-literal meaning.
Mutual information: A measure of the statistical dependence between two signals.
Syntactic prose sentence: A sentence that is syntactically correct but semantically incoherent.

## References

[bib1] Ahissar, E., Nagarajan, S., Ahissar, M., Protopapas, A., Mahncke, H., & Merzenich, M. M. (2001). Speech comprehension is correlated with temporal response patterns recorded from auditory cortex. Proceedings of the National Academy of Sciences, 98(23), 13367–13372. 10.1073/pnas.201400998, 11698688 PMC60877

[bib2] Baayen, R. H., Davidson, D. J., & Bates, D. M. (2008). Mixed-effects modeling with crossed random effects for subjects and items. Journal of Memory and Language, 59(4), 390–412. 10.1016/j.jml.2007.12.005

[bib3] Baggio, G. (2021). Compositionality in a parallel architecture for language processing. Cognitive Science, 45(5), Article e12949. 10.1111/cogs.12949, 34018238

[bib4] Baggio, G., & Hagoort, P. (2011). The balance between memory and unification in semantics: A dynamic account of the N400. Language and Cognitive Processes, 26(9), 1338–1367. 10.1080/01690965.2010.542671

[bib5] Bastiaansen, M. C. M., & Hagoort, P. (2015). Frequency-based segregation of syntactic and semantic unification during online sentence level language comprehension. Journal of Cognitive Neuroscience, 27(11), 2095–2107. 10.1162/jocn_a_00829, 26042498

[bib6] Bates, D., Mächler, M., Bolker, B., & Walker, S. (2015). Fitting linear mixed-effects models using lme4. Journal of Statistical Software, 67(1), 1–48. 10.18637/jss.v067.i01

[bib7] Blanco-Elorrieta, E., Ding, N., Pylkkänen, L., & Poeppel, D. (2020). Understanding requires tracking: Noise and knowledge interact in bilingual comprehension. Journal of Cognitive Neuroscience, 32(10), 1975–1983. 10.1162/jocn_a_01610, 32662732

[bib8] Boersma, P., & Weenink, D. (2019). Praat: Doing phonetics by computer (Version 6.1.02) [Computer software]. https://www.fon.hum.uva.nl/praat/

[bib9] Bourguignon, M., De Tiège, X., Op De Beeck, M., Ligot, N., Paquier, P., Van Bogaert, P., Goldman, S., Hari, R., & Jousmäki, V. (2013). The pace of prosodic phrasing couples the listener’s cortex to the reader’s voice. Human Brain Mapping, 34(2), 314–326. 10.1002/hbm.21442, 22392861 PMC6869855

[bib11] Brennan, J. R., & Martin, A. E. (2020). Phase synchronization varies systematically with linguistic structure composition. Philosophical Transactions of the Royal Society B: Biological Sciences, 375(1791), Article 20190305. 10.1098/rstb.2019.0305, 31840584 PMC6939345

[bib10] Brennan, J. [R.], Nir, Y., Hasson, U., Malach, R., Heeger, D. J., & Pylkkänen, L. (2012). Syntactic structure building in the anterior temporal lobe during natural story listening. Brain and Language, 120(2), 163–173. 10.1016/j.bandl.2010.04.002, 20472279 PMC2947556

[bib12] Brennan, J. R., Stabler, E. P., Van Wagenen, S. E., Luh, W.-M., & Hale, J. T. (2016). Abstract linguistic structure correlates with temporal activity during naturalistic comprehension. Brain and Language, 157–158, 81–94. 10.1016/j.bandl.2016.04.008, 27208858 PMC4893969

[bib13] Brodbeck, C., Presacco, A., & Simon, J. Z. (2018). Neural source dynamics of brain responses to continuous stimuli: Speech processing from acoustics to comprehension. NeuroImage, 172, 162–174. 10.1016/j.neuroimage.2018.01.042, 29366698 PMC5910254

[bib14] Broderick, M. P., Anderson, A. J., & Lalor, E. C. (2019). Semantic context enhances the early auditory encoding of natural speech. Journal of Neuroscience, 39(38), 7564–7575. 10.1523/JNEUROSCI.0584-19.2019, 31371424 PMC6750931

[bib15] Burroughs, A., Kazanina, N., & Houghton, C. (2021). Grammatical category and the neural processing of phrases. Scientific Reports, 11(1), Article 2446. 10.1038/s41598-021-81901-5, 33510230 PMC7844293

[bib16] Cacciari, C. (2014). Processing multiword idiomatic strings: Many words in one? The Mental Lexicon, 9(2), 267–293. 10.1075/ml.9.2.05cac

[bib17] Cacciari, C., & Corradini, P. (2015). Literal analysis and idiom retrieval in ambiguous idioms processing: A reading-time study. Journal of Cognitive Psychology, 27(7), 797–811. 10.1080/20445911.2015.1049178

[bib18] Cacciari, C., & Glucksberg, S. (1991). Understanding idiomatic expressions: The contribution of word meanings. In G. B. Simpson (Ed.), Understanding word and sentence (pp. 217–240). Elsevier. 10.1016/S0166-4115(08)61535-6

[bib19] Cacciari, C., Padovani, R., & Corradini, P. (2007). Exploring the relationship between individuals’ speed of processing and their comprehension of spoken idioms. European Journal of Cognitive Psychology, 19(3), 417–445. 10.1080/09541440600763705

[bib20] Cacciari, C., & Tabossi, P. (1988). The comprehension of idioms. Journal of Memory and Language, 27(6), 668–683. 10.1016/0749-596X(88)90014-9

[bib21] Canal, P., Pesciarelli, F., Vespignani, F., Molinaro, N., & Cacciari, C. (2017). Basic composition and enriched integration in idiom processing: An EEG study. Journal of Experimental Psychology: Learning, Memory, and Cognition, 43(6), 928–943. 10.1037/xlm0000351, 28068127

[bib22] Chandrasekaran, C., Trubanova, A., Stillittano, S., Caplier, A., & Ghazanfar, A. A. (2009). The natural statistics of audiovisual speech. PLOS Computational Biology, 5(7), Article e1000436. 10.1371/journal.pcbi.1000436, 19609344 PMC2700967

[bib23] Chomsky, N. (1957). Syntactic structures. Mouton. 10.1515/9783112316009

[bib24] Cogan, G. B., & Poeppel, D. (2011). A mutual information analysis of neural coding of speech by low-frequency MEG phase information. Journal of Neurophysiology, 106(2), 554–563. 10.1152/jn.00075.2011, 21562190 PMC3154802

[bib25] Coopmans, C. W., & Nieuwland, M. S. (2020). Dissociating activation and integration of discourse referents: Evidence from ERPs and oscillations. Cortex, 126, 83–106. 10.1016/j.cortex.2019.12.028, 32065957

[bib26] Cutting, J. C., & Bock, K. (1997). That’s the way the cookie bounces: Syntactic and semantic components of experimentally elicited idiom blends. Memory & Cognition, 25(1), 57–71. 10.3758/BF03197285, 9046870

[bib27] Di Liberto, G. M., Lalor, E. C., & Millman, R. E. (2018). Causal cortical dynamics of a predictive enhancement of speech intelligibility. NeuroImage, 166, 247–258. 10.1016/j.neuroimage.2017.10.066, 29102808

[bib28] Ding, N., Melloni, L., Yang, A., Wang, Y., Zhang, W., & Poeppel, D. (2017). Characterizing neural entrainment to hierarchical linguistic units using electroencephalography (EEG). Frontiers in Human Neuroscience, 11, Article 481. 10.3389/fnhum.2017.00481, 29033809 PMC5624994

[bib29] Ding, N., Melloni, L., Zhang, H., Tian, X., & Poeppel, D. (2016). Cortical tracking of hierarchical linguistic structures in connected speech. Nature Neuroscience, 19(1), 158–164. 10.1038/nn.4186, 26642090 PMC4809195

[bib30] Ding, N., Patel, A. D., Chen, L., Butler, H., Luo, C., & Poeppel, D. (2017). Temporal modulations in speech and music. Neuroscience & Biobehavioral Reviews, 81(Pt. B), 181–187. 10.1016/j.neubiorev.2017.02.011, 28212857

[bib31] Ding, N., & Simon, J. Z. (2014). Cortical entrainment to continuous speech: Functional roles and interpretations. Frontiers in Human Neuroscience, 8, Article 311. 10.3389/fnhum.2014.00311, 24904354 PMC4036061

[bib32] Doelling, K. B., Arnal, L. H., Ghitza, O., & Poeppel, D. (2014). Acoustic landmarks drive delta–theta oscillations to enable speech comprehension by facilitating perceptual parsing. NeuroImage, 85(Pt. 2), 761–768. 10.1016/j.neuroimage.2013.06.035, 23791839 PMC3839250

[bib33] Donhauser, P. W., & Baillet, S. (2020). Two distinct neural timescales for predictive speech processing. Neuron, 105(2), 385–393. 10.1016/j.neuron.2019.10.019, 31806493 PMC6981026

[bib34] Fedorenko, E., Blank, I. A., Siegelman, M., & Mineroff, Z. (2020). Lack of selectivity for syntax relative to word meanings throughout the language network. Cognition, 203, Article 104348. 10.1016/j.cognition.2020.104348, 32569894 PMC7483589

[bib35] Fedorenko, E., Scott, T. L., Brunner, P., Coon, W. G., Pritchett, B., Schalk, G., & Kanwisher, N. (2016). Neural correlate of the construction of sentence meaning. Proceedings of the National Academy of Sciences, 113(41), E6256–E6262. 10.1073/pnas.1612132113, 27671642 PMC5068329

[bib36] Frank, S. L., & Yang, J. (2018). Lexical representation explains cortical entrainment during speech comprehension. PLOS One, 13(5), Article e0197304. 10.1371/journal.pone.0197304, 29771964 PMC5957381

[bib37] Getz, H., Ding, N., Newport, E. L., & Poeppel, D. (2018). Cortical tracking of constituent structure in language acquisition. Cognition, 181, 135–140. 10.1016/j.cognition.2018.08.019, 30195135 PMC6201233

[bib38] Ghitza, O. (2017). Acoustic-driven delta rhythms as prosodic markers. Language, Cognition and Neuroscience, 32(5), 545–561. 10.1080/23273798.2016.1232419

[bib39] Gibbs, R. W., Nayak, N. P., & Cutting, C. (1989). How to kick the bucket and not decompose: Analyzability and idiom processing. Journal of Memory and Language, 28(5), 576–593. 10.1016/0749-596X(89)90014-4

[bib40] Giraud, A.-L., & Poeppel, D. (2012). Cortical oscillations and speech processing: Emerging computational principles and operations. Nature Neuroscience, 15(4), 511–517. 10.1038/nn.3063, 22426255 PMC4461038

[bib41] Gross, J., Hoogenboom, N., Thut, G., Schyns, P., Panzeri, S., Belin, P., & Garrod, S. (2013). Speech rhythms and multiplexed oscillatory sensory coding in the human brain. PLOS Biology, 11(12), Article e1001752. 10.1371/journal.pbio.1001752, 24391472 PMC3876971

[bib42] Hagoort, P. (2005). On Broca, brain, and binding: A new framework. Trends in Cognitive Sciences, 9(9), 416–423. 10.1016/j.tics.2005.07.004, 16054419

[bib43] Hagoort, P. (2017). The core and beyond in the language-ready brain. Neuroscience & Biobehavioral Reviews, 81(Pt. B), 194–204. 10.1016/j.neubiorev.2017.01.048, 28193452

[bib44] Holsinger, E., & Kaiser, E. (2013). Processing (non)compositional expressions: Mistakes and recovery. Journal of Experimental Psychology: Learning, Memory, and Cognition, 39(3), 866–878. 10.1037/a0030410, 23088547

[bib45] Howard, M. F., & Poeppel, D. (2010). Discrimination of speech stimuli based on neuronal response phase patterns depends on acoustics but not comprehension. Journal of Neurophysiology, 104(5), 2500–2511. 10.1152/jn.00251.2010, 20484530 PMC2997028

[bib46] Hubers, F., Cucchiarini, C., Strik, H., & Dijkstra, T. (2021). Individual word activation and word frequency effects during the processing of opaque idiomatic expressions. Quarterly Journal of Experimental Psychology, 1–17. 10.1177/17470218211047995, 34507505 PMC9016674

[bib47] Hubers, F. C. W., van Ginkel, W., Cucchiarini, C., Strik, H., & Dijkstra, A. F. J. (2018). Normative data on Dutch idiomatic expressions: Native speakers [Data set]. Data Archiving and Networked Services (DANS). 75(6). 10.17026/dans-zjx-hnsk

[bib48] Ince, R. A. A., Giordano, B. L., Kayser, C., Rousselet, G. A., Gross, J., & Schyns, P. G. (2017). A statistical framework for neuroimaging data analysis based on mutual information estimated via a Gaussian copula. Human Brain Mapping, 38(3), 1541–1573. 10.1002/hbm.23471, 27860095 PMC5324576

[bib49] Jackendoff, R. (1995). The boundaries of the lexicon. In M. Everaert, E. J. van der Linden, A. Schenk, & R. Schroeder (Eds.), Idioms: Structural and psychological perspectives (pp. 133–165). Erlbaum.

[bib50] Jackendoff, R. (2017). In defense of theory. Cognitive Science, 41(S2), 185–212. 10.1111/cogs.12324, 26611772

[bib51] Jin, P., Lu, Y., & Ding, N. (2020). Low-frequency neural activity reflects rule-based chunking during speech listening. eLife, 9, Article e55613. 10.7554/eLife.55613, 32310082 PMC7213976

[bib52] Kaan, E., & Swaab, T. Y. (2002). The brain circuitry of syntactic comprehension. Trends in Cognitive Sciences, 6(8), 350–356. 10.1016/S1364-6613(02)01947-2, 12140086

[bib53] Kaufeld, G., Bosker, H. R., ten Oever, S., Alday, P. M., Meyer, A. S., & Martin, A. E. (2020). Linguistic structure and meaning organize neural oscillations into a content-specific hierarchy. Journal of Neuroscience, 40(49), 9467–9475. 10.1523/JNEUROSCI.0302-20.2020, 33097640 PMC7724143

[bib54] Kayser, S. J., Ince, R. A. A., Gross, J., & Kayser, C. (2015). Irregular speech rate dissociates auditory cortical entrainment, evoked responses, and frontal alpha. Journal of Neuroscience, 35(44), 14691–14701. 10.1523/JNEUROSCI.2243-15.2015, 26538641 PMC4635123

[bib55] Keitel, A., Gross, J., & Kayser, C. (2018). Perceptually relevant speech tracking in auditory and motor cortex reflects distinct linguistic features. PLOS Biology, 16(3), Article e2004473. 10.1371/journal.pbio.2004473, 29529019 PMC5864086

[bib56] Keitel, A., Ince, R. A. A., Gross, J., & Kayser, C. (2017). Auditory cortical delta-entrainment interacts with oscillatory power in multiple fronto-parietal networks. NeuroImage, 147, 32–42. 10.1016/j.neuroimage.2016.11.062, 27903440 PMC5315055

[bib57] Keuleers, E., & Brysbaert, M. (2010). Wuggy: A multilingual pseudoword generator. Behavior Research Methods, 42(3), 627–633. 10.3758/BRM.42.3.627, 20805584

[bib58] Keuleers, E., Brysbaert, M., & New, B. (2010). SUBTLEX-NL: A new measure for Dutch word frequency based on film subtitles. Behavior Research Methods, 42(3), 643–650. 10.3758/BRM.42.3.643, 20805586

[bib59] Konopka, A. E., & Bock, K. (2009). Lexical or syntactic control of sentence formulation? Structural generalizations from idiom production. Cognitive Psychology, 58(1), 68–101. 10.1016/j.cogpsych.2008.05.002, 18644587

[bib60] Kösem, A., & van Wassenhove, V. (2017). Distinct contributions of low- and high-frequency neural oscillations to speech comprehension. Language, Cognition and Neuroscience, 32(5), 536–544. 10.1080/23273798.2016.1238495

[bib61] Kutas, M., & Federmeier, K. D. (2011). Thirty years and counting: Finding meaning in the N400 component of the event-related brain potential (ERP). Annual Review of Psychology, 62(1), 621–647. 10.1146/annurev.psych.093008.131123, 20809790 PMC4052444

[bib62] Libben, M. R., & Titone, D. A. (2008). The multidetermined nature of idiom processing. Memory & Cognition, 36(6), 1103–1121. 10.3758/MC.36.6.1103, 18927029

[bib63] Luo, H., & Poeppel, D. (2007). Phase patterns of neuronal responses reliably discriminate speech in human auditory cortex. Neuron, 54(6), 1001–1010. 10.1016/j.neuron.2007.06.004, 17582338 PMC2703451

[bib64] Mai, G., Minett, J. W., & Wang, W. S.-Y. (2016). Delta, theta, beta, and gamma brain oscillations index levels of auditory sentence processing. NeuroImage, 133, 516–528. 10.1016/j.neuroimage.2016.02.064, 26931813

[bib65] Makov, S., Sharon, O., Ding, N., Ben-Shachar, M., Nir, Y., & Golumbic, E. Z. (2017). Sleep disrupts high-level speech parsing despite significant basic auditory processing. Journal of Neuroscience, 37(32), 7772–7781. 10.1523/JNEUROSCI.0168-17.2017, 28626013 PMC6596654

[bib66] Marslen-Wilson, W. D. (2007). Morphological processes in language comprehension. In M. G. Gaskell (Ed.), Oxford handbook of psycholinguistics (pp. 175–186). Oxford University Press. 10.1093/oxfordhb/9780198568971.013.0011

[bib67] Marslen-Wilson, W. [D.], & Tyler, L. K. (1980). The temporal structure of spoken language understanding. Cognition, 8(1), 1–71. 10.1016/0010-0277(80)90015-3, 7363578

[bib68] Martin, A. E. (2016). Language processing as cue integration: Grounding the psychology of language in perception and neurophysiology. Frontiers in Psychology, 7, Article 120. 10.3389/fpsyg.2016.00120, 26909051 PMC4754405

[bib69] Martin, A. E. (2020). A compositional neural architecture for language. Journal of Cognitive Neuroscience, 32(8), 1407–1427. 10.1162/jocn_a_01552, 32108553

[bib70] Martin, A. E., & Doumas, L. A. A. (2017). A mechanism for the cortical computation of hierarchical linguistic structure. PLOS Biology, 15(3), Article e2000663. 10.1371/journal.pbio.2000663, 28253256 PMC5333798

[bib71] Martin, A. E., & Doumas, L. A. A. (2019). Predicate learning in neural systems: Using oscillations to discover latent structure. Current Opinion in Behavioral Sciences, 29, 77–83. 10.1016/j.cobeha.2019.04.008

[bib72] Matchin, W., Brodbeck, C., Hammerly, C., & Lau, E. (2019). The temporal dynamics of structure and content in sentence comprehension: Evidence from fMRI-constrained MEG. Human Brain Mapping, 40(2), 663–678. 10.1002/hbm.24403, 30259599 PMC6865621

[bib73] Matchin, W., & Hickok, G. (2020). The cortical organization of syntax. Cerebral Cortex, 30(3), 1481–1498. 10.1093/cercor/bhz180, 31670779 PMC7132936

[bib74] Mazoyer, B. M., Tzourio, N., Frak, V., Syrota, A., Murayama, N., Levrier, O., Salamon, G., Dehaene, S., Cohen, L., & Mehler, J. (1993). The cortical representation of speech. Journal of Cognitive Neuroscience, 5(4), 467–479. 10.1162/jocn.1993.5.4.467, 23964919

[bib75] Meyer, L., Henry, M. J., Gaston, P., Schmuck, N., & Friederici, A. D. (2017). Linguistic bias modulates interpretation of speech via neural delta-band oscillations. Cerebral Cortex, 27(9), 4293–4302. 10.1093/cercor/bhw228, 27566979

[bib76] Meyer, L., Sun, Y., & Martin, A. E. (2020). Synchronous, but not entrained: Exogenous and endogenous cortical rhythms of speech and language processing. Language, Cognition and Neuroscience, 35(9), 1089–1099. 10.1080/23273798.2019.1693050

[bib77] Molinaro, N., & Lizarazu, M. (2018). Delta(but not theta)-band cortical entrainment involves speech-specific processing. European Journal of Neuroscience, 48(7), 2642–2650. 10.1111/ejn.13811, 29283465

[bib78] Molinaro, N., Lizarazu, M., Baldin, V., Pérez-Navarro, J., Lallier, M., & Ríos-López, P. (2021). Speech-brain phase coupling is enhanced in low contextual semantic predictability conditions. Neuropsychologia, 156, Article 107830. 10.1016/j.neuropsychologia.2021.107830, 33771540

[bib79] Mollica, F., Siegelman, M., Diachek, E., Piantadosi, S. T., Mineroff, Z., Futrell, R., Kean, H., Qian, P., & Fedorenko, E. (2020). Composition is the core driver of the language-selective network. Neurobiology of Language, 1(1), 104–134. 10.1162/nol_a_00005 36794007 PMC9923699

[bib80] Moreno, E. M., Federmeier, K. D., & Kutas, M. (2002). Switching languages, switching palabras (words): An electrophysiological study of code switching. Brain and Language, 80(2), 188–207. 10.1006/brln.2001.2588, 11827443

[bib81] Nelson, M. J., El Karoui, I., Giber, K., Yang, X., Cohen, L., Koopman, H., Cash, S. S., Naccache, L., Hale, J. T., Pallier, C., & Dehaene, S. (2017). Neurophysiological dynamics of phrase-structure building during sentence processing. Proceedings of the National Academy of Sciences, 114(18), E3669–E3678. 10.1073/pnas.1701590114, 28416691 PMC5422821

[bib82] Obleser, J., & Kayser, C. (2019). Neural entrainment and attentional selection in the listening brain. Trends in Cognitive Sciences, 23(11), 913–926. 10.1016/j.tics.2019.08.004, 31606386

[bib83] Oostenveld, R., Fries, P., Maris, E., & Schoffelen, J.-M. (2011). FieldTrip: Open source software for advanced analysis of MEG, EEG, and invasive electrophysiological data. Computational Intelligence and Neuroscience, 2011, Article 156869. 10.1155/2011/156869, 21253357 PMC3021840

[bib84] Park, H., Ince, R. A. A., Schyns, P. G., Thut, G., & Gross, J. (2015). Frontal top-down signals increase coupling of auditory low-frequency oscillations to continuous speech in human listeners. Current Biology, 25(12), 1649–1653. 10.1016/j.cub.2015.04.049, 26028433 PMC4503802

[bib85] Peelle, J. E., & Davis, M. H. (2012). Neural oscillations carry speech rhythm through to comprehension. Frontiers in Psychology, 3, Article 320. 10.3389/fpsyg.2012.00320, 22973251 PMC3434440

[bib86] Peña, M., & Melloni, L. (2012). Brain oscillations during spoken sentence processing. Journal of Cognitive Neuroscience, 24(5), 1149–1164. 10.1162/jocn_a_00144, 21981666

[bib87] Peterson, R. R., Burgess, C., Dell, G. S., & Eberhard, K. M. (2001). Dissociation between syntactic and semantic processing during idiom comprehension. Journal of Experimental Psychology: Learning, Memory, and Cognition, 27(5), 1223–1237. 10.1037/0278-7393.27.5.1223, 11550750

[bib88] Poeppel, D., & Assaneo, M. F. (2020). Speech rhythms and their neural foundations. Nature Reviews Neuroscience, 21(6), 322–334. 10.1038/s41583-020-0304-4, 32376899

[bib89] Post, B., Marslen-Wilson, W. D., Randall, B., & Tyler, L. K. (2008). The processing of English regular inflections: Phonological cues to morphological structure. Cognition, 109(1), 1–17. 10.1016/j.cognition.2008.06.011, 18834584 PMC2596971

[bib90] R Core Team. (2021). R: A language and environment for statistical computing [Computer software]. R Foundation for Statistical Computing. https://www.r-project.org

[bib91] Rimmele, J. M., Morillon, B., Poeppel, D., & Arnal, L. H. (2018). Proactive sensing of periodic and aperiodic auditory patterns. Trends in Cognitive Sciences, 22(10), 870–882. 10.1016/j.tics.2018.08.003, 30266147

[bib92] Rimmele, J. M., Poeppel, D., & Ghitza, O. (2021). Acoustically driven cortical δ oscillations underpin prosodic chunking. Eneuro, 8(4), Article 0562-20.2021. 10.1523/ENEURO.0562-20.2021, 34083380 PMC8272402

[bib93] Rommers, J., Dijkstra, T., & Bastiaansen, M. (2013). Context-dependent semantic processing in the human brain: Evidence from idiom comprehension. Journal of Cognitive Neuroscience, 25(5), 762–776. 10.1162/jocn_a_00337, 23249356

[bib94] Schell, M., Zaccarella, E., & Friederici, A. D. (2017). Differential cortical contribution of syntax and semantics: An fMRI study on two-word phrasal processing. Cortex, 96, 105–120. 10.1016/j.cortex.2017.09.002, 29024818

[bib95] Schroeder, C. E., & Lakatos, P. (2009). Low-frequency neuronal oscillations as instruments of sensory selection. Trends in Neurosciences, 32(1), 9–18. 10.1016/j.tins.2008.09.012, 19012975 PMC2990947

[bib96] Sheng, J., Zheng, L., Lyu, B., Cen, Z., Qin, L., Tan, L. H., Huang, M.-X., Ding, N., & Gao, J.-H. (2019). The cortical maps of hierarchical linguistic structures during speech perception. Cerebral Cortex, 29(8), 3232–3240. 10.1093/cercor/bhy191, 30137249

[bib97] Smith, Z. M., Delgutte, B., & Oxenham, A. J. (2002). Chimaeric sounds reveal dichotomies in auditory perception. Nature, 416(6876), 87–90. 10.1038/416087a, 11882898 PMC2268248

[bib98] Smolka, E., Rabanus, S., & Rösler, F. (2007). Processing verbs in German idioms: Evidence against the configuration hypothesis. Metaphor and Symbol, 22(3), 213–231. 10.1080/10926480701357638

[bib99] Sprenger, S. A., Levelt, W. J. M., & Kempen, G. (2006). Lexical access during the production of idiomatic phrases. Journal of Memory and Language, 54(2), 161–184. 10.1016/j.jml.2005.11.001

[bib100] ten Oever, S., & Martin, A. E. (2021). An oscillating computational model can track pseudo-rhythmic speech by using linguistic predictions. eLife, 10, Article e68066. 10.7554/eLife.68066, 34338196 PMC8328513

[bib101] Titone, D. A., & Connine, C. M. (1999). On the compositional and noncompositional nature of idiomatic expressions. Journal of Pragmatics, 31(12), 1655–1674. 10.1016/S0378-2166(99)00008-9

[bib102] Tyler, L. K., Stamatakis, E. A., Post, B., Randall, B., & Marslen-Wilson, W. (2005). Temporal and frontal systems in speech comprehension: An fMRI study of past tense processing. Neuropsychologia, 43(13), 1963–1974. 10.1016/j.neuropsychologia.2005.03.008, 16168736

[bib103] Vespignani, F., Canal, P., Molinaro, N., Fonda, S., & Cacciari, C. (2010). Predictive mechanisms in idiom comprehension. Journal of Cognitive Neuroscience, 22(8), 1682–1700. 10.1162/jocn.2009.21293, 19580384

[bib104] Westerlund, M., & Pylkkänen, L. (2014). The role of the left anterior temporal lobe in semantic composition vs. semantic memory. Neuropsychologia, 57, 59–70. 10.1016/j.neuropsychologia.2014.03.001, 24631260

[bib105] Zhang, L., & Pylkkänen, L. (2015). The interplay of composition and concept specificity in the left anterior temporal lobe: An MEG study. NeuroImage, 111, 228–240. 10.1016/j.neuroimage.2015.02.028, 25703829

[bib106] Zoefel, B., & VanRullen, R. (2015). The role of high-level processes for oscillatory phase entrainment to speech sound. Frontiers in Human Neuroscience, 9, Article 651. 10.3389/fnhum.2015.00651, 26696863 PMC4667100

[bib107] Zoefel, B., & VanRullen, R. (2016). EEG oscillations entrain their phase to high-level features of speech sound. NeuroImage, 124, 16–23. 10.1016/j.neuroimage.2015.08.054, 26341026

[bib108] Zou, J., Feng, J., Xu, T., Jin, P., Luo, C., Zhang, J., Pan, X., Chen, F., Zheng, J., & Ding, N. (2019). Auditory and language contributions to neural encoding of speech features in noisy environments. NeuroImage, 192, 66–75. 10.1016/j.neuroimage.2019.02.047, 30822469

