# Supplementary material for: Effects of Structure and Meaning on Cortical Tracking of Linguistic Units in Naturalistic Speech
Source: Neurobiol Lang (Camb). 2022 Jun 21;3(3):386–412. doi: 10.1162/nol_a_00070 (PMC10158633; doi:10.1162/nol_a_00070)
Supplement: Supplementary file 1 [file nol-3-3-386-s001.docx]

**Supplementary Information for**

**Effects of structure and meaning on cortical tracking of linguistic units in naturalistic speech**

**S1. Analysis of modulation spectra**

Figure S1A shows the modulation spectra of all forward conditions, computed following the procedure described in Ding, Patel, Chen, Butler, Luo, and Poeppel (2017). For each condition we concatenated all recordings, cut the long sound recording into 4-s duration segments, and then calculated the modulation spectrum of each segment separately. Figure S1A shows the modulation spectra after averaging over segments. The Word list condition visibly deviates from the other forward conditions, which are otherwise very similar. To quantify the difference between the modulation spectra of Sentences and the modulation spectra of the other conditions, we computed the area under the curve (AUC) of the modulation spectrum of each segment of each condition and compared the AUCs across conditions.

The distribution of the resulting AUCs are presented in the probability density plot in Figure S1B. Comparison of the five groups (Sentence, Idiom, Syntactic Prose, Jabberwocky, Word list) through a one-way ANOVA in R (R Core Team, 2021) indeed reveals that the AUCs between groups were different, *F*(3,267) = 17.2, *p* < .001. Pair-wise follow-up tests show that only the AUCs for Word lists and Sentences differed, Δ = -2.72, 95% CI [-3.94, -1.49], p < .001. The AUCs for Sentences did not differ from the AUCs for Idioms (Δ = -0.44, 95% CI [-1.71, 0.92], p = .87), Jabberwocky (Δ = 0.067, 95% CI [-1.20 1.34], p = 1.00), or Syntactic prose (Δ = 0.49, 95% CI [-0.79, 1.78], p = .83). Given the difference between the recordings for Sentences and Word lists, we included two conditions to control for these acoustic differences. One control condition contained backward versions of the Sentence recordings (i.e., each recording time-reversed), and the other contained backward versions of the Word list recordings.

**
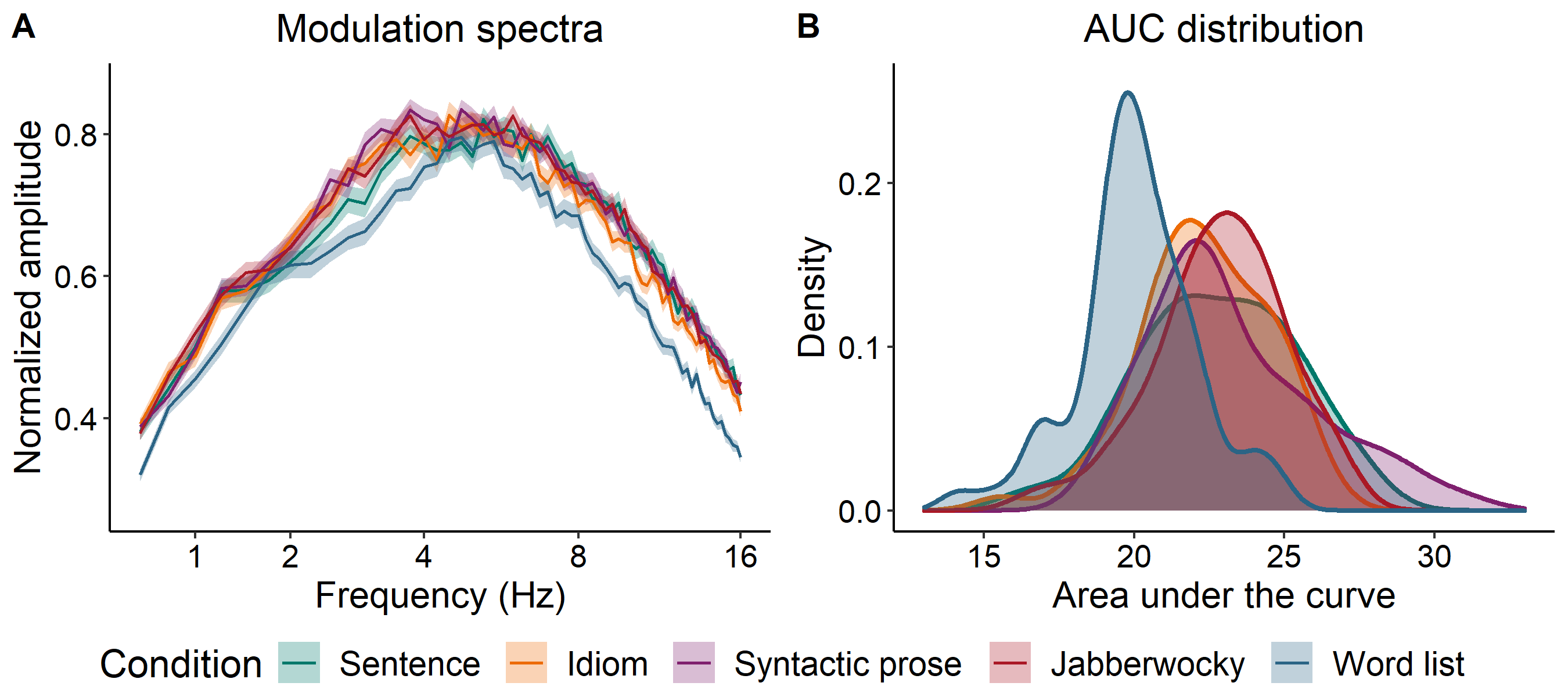
**

**Figure S1.** **(A)** Modulation spectra of the forward versions of all conditions. The Word list condition is visibly different from the sentence condition. **(B)** Probability density plots representing the distribution of the areas under the curve (AUC) for the modulation spectra of all forward recordings, which again show the difference between the Word list and the Sentence condition.


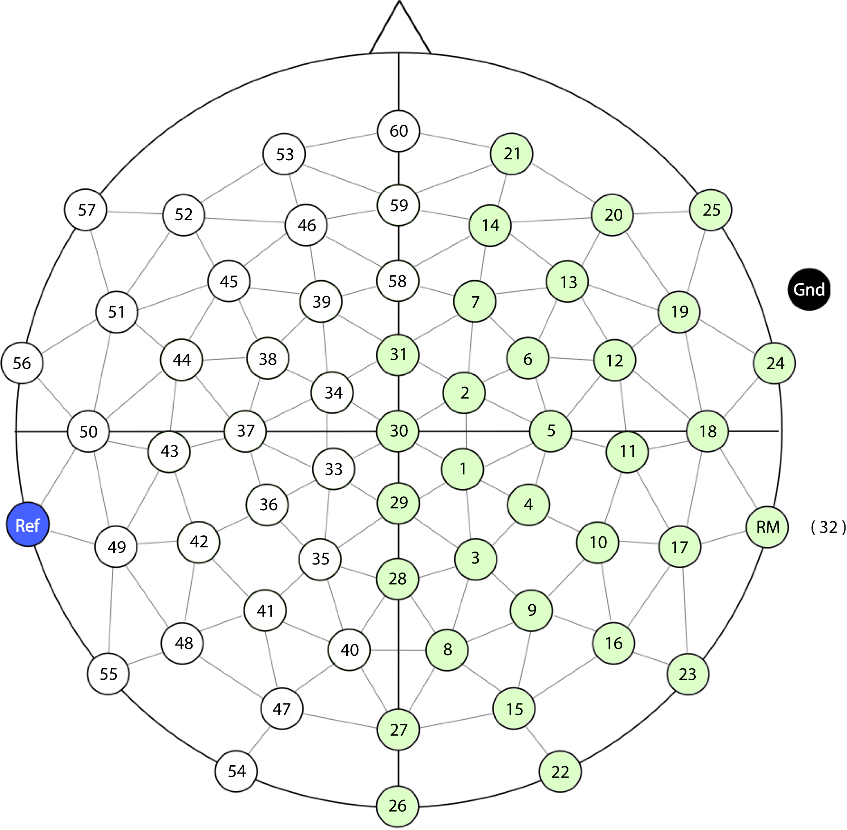
**S2. EEG electrode layout**

**Figure S2.** Schematic representation of the 59-electrode array layout.

**S3. Spectral power analysis**

We did not find differences in speech tracking between Sentences and Word lists, which might be related to the acoustic differences between their audio recordings (see Figure S1). As an exploratory analysis, we examined whether any differences between these conditions could be found in spectral power in the frequency band corresponding to phrases (see e.g., Bonhage, Meyer, Gruber, Friederici, & Mueller, 2017; Ding, Melloni, Zhang, Tian, & Poeppel, 2016). In contrast to the analysis of speech tracking, this analysis is not based on a comparison of the brain signal and the actual audio signal and might therefore be less affected by acoustic differences between these audio signals.

The topographies in Figure S3 reflect the grand average spectral power in the phrase frequency band (1-2 Hz), derived by a fast Fourier transform (Hanning window) of each EEG epoch (all zero-padded to 4 seconds). Delta power in all conditions, including the two backward conditions, has a bilateral distribution. We compared these effects using cluster-based random permutation tests (Maris & Oostenveld, 2007) in Fieldtrip (Oostenveld, Fries, Maris, & Schoffelen, 2011). These analyses showed effects of direction for both structures. That is, Forward Sentences elicited stronger delta power Backward Sentences (one positive cluster, p = .004) and Forward Word lists elicited stronger delta power Backward Word lists (one positive cluster, p = .002), but these effects of direction were not different across structures (i.e., no interaction).


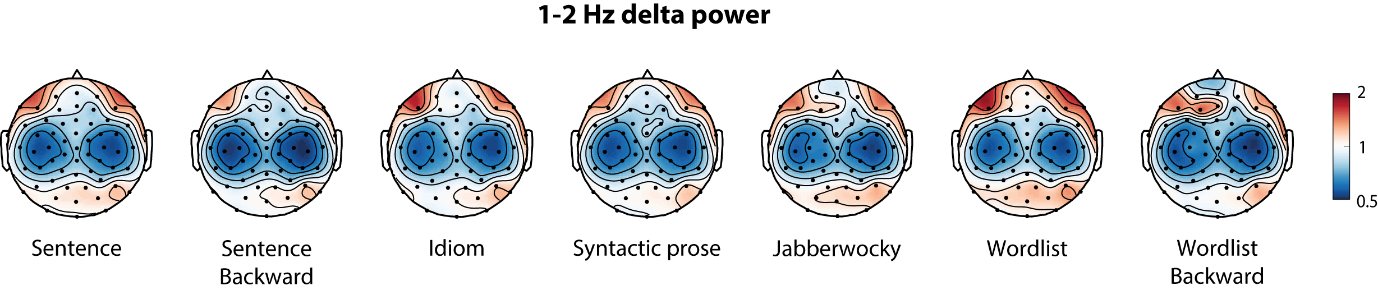


**Figure S3.** Topographical plots of 1-2 Hz delta power in each condition.

**References**

Bonhage, C. E., Meyer, L., Gruber, T., Friederici, A. D., & Mueller, J. L. (2017). Oscillatory EEG dynamics underlying automatic chunking during sentence processing. *NeuroImage*, *152*, 647– 657. https://doi.org/10.1016/j.neuroimage.2017.03.018

Ding, N., Melloni, L., Zhang, H., Tian, X., & Poeppel, D. (2016). Cortical tracking of hierarchical linguistic structures in connected speech. *Nature Neuroscience*, *19*(1), 158–164. https://doi.org/10.1038/nn.4186

Ding, N., Patel, A. D., Chen, L., Butler, H., Luo, C., & Poeppel, D. (2017). Temporal modulations in speech and music. *Neuroscience & Biobehavioral Reviews*, *81*, 181–187. https://doi.org/10.1016/j.neubiorev.2017.02.011

Maris, E., & Oostenveld, R. (2007). Nonparametric statistical testing of EEG- and MEG-data. *Journal of Neuroscience Methods*, *164*(1), 177–190. https://doi.org/10.1016/j.jneumeth.2007.03.024

Oostenveld, R., Fries, P., Maris, E., & Schoffelen, J.-M. (2011). FieldTrip: Open source software for advanced analysis of MEG, EEG, and invasive electrophysiological data. *Computational Intelligence and Neuroscience*, *156869*. https://doi.org/10.1155/2011/156869

R Core Team. (2021). *R: A language and environment for statistical computing****.*** Vienna, Austria: R Foundation for Statistical Computing. https://www.r-project.org
